# Integration of estimated regional gene expression with neuroimaging and clinical phenotypes at biobank scale

**DOI:** 10.1371/journal.pbio.3002782

**Published:** 2024-09-13

**Authors:** Nhung Hoang, Neda Sardaripour, Grace D. Ramey, Kurt Schilling, Emily Liao, Yiting Chen, Jee Hyun Park, Xavier Bledsoe, Bennett A. Landman, Eric R. Gamazon, Mary Lauren Benton, John A. Capra, Mikail Rubinov

**Affiliations:** 1 Department of Computer Science, Vanderbilt University, Nashville, Tennessee, United States of America; 2 Department of Biomedical Engineering, Vanderbilt University, Nashville, Tennessee, United States of America; 3 Biological and Medical Informatics Division, University of California, San Francisco, California, United States of America; 4 Department of Epidemiology and Biostatistics, University of California, San Francisco, California, United States of America; 5 Department of Electrical and Computer Engineering, Vanderbilt University, Nashville, Tennessee, United States of America; 6 Department of Radiology and Radiological Sciences, Vanderbilt University Medical Center, Nashville, Tennessee, United States of America; 7 Vanderbilt Genetics Institute, Vanderbilt University Medical Center, Nashville, Tennessee, United States of America; 8 Department of Computer Science, Baylor University, Waco, Texas, United States of America; 9 Department of Biological Sciences, Vanderbilt University, Nashville, Tennessee, United States of America; 10 Bakar Computational Health Sciences Institute, University of California, San Francisco, California, United States of America; 11 Department of Psychology, Vanderbilt University, Nashville, Tennessee, United States of America; 12 Howard Hughes Medical Institute Janelia Research Campus, Ashburn, Virginia, United States of America; The University of Melbourne, AUSTRALIA

## Abstract

An understanding of human brain individuality requires the integration of data on brain organization across people and brain regions, molecular and systems scales, as well as healthy and clinical states. Here, we help advance this understanding by leveraging methods from computational genomics to integrate large-scale genomic, transcriptomic, neuroimaging, and electronic-health record data sets. We estimated genetically regulated gene expression (gr-expression) of 18,647 genes, across 10 cortical and subcortical regions of 45,549 people from the UK Biobank. First, we showed that patterns of estimated gr-expression reflect known genetic–ancestry relationships, regional identities, as well as inter-regional correlation structure of directly assayed gene expression. Second, we performed transcriptome-wide association studies (TWAS) to discover 1,065 associations between individual variation in gr-expression and gray-matter volumes across people and brain regions. We benchmarked these associations against results from genome-wide association studies (GWAS) of the same sample and found hundreds of novel associations relative to these GWAS. Third, we integrated our results with clinical associations of gr-expression from the Vanderbilt Biobank. This integration allowed us to link genes, via gr-expression, to neuroimaging and clinical phenotypes. Fourth, we identified associations of polygenic gr-expression with structural and functional MRI phenotypes in the Human Connectome Project (HCP), a small neuroimaging-genomic data set with high-quality functional imaging data. Finally, we showed that estimates of gr-expression and magnitudes of TWAS were generally replicable and that the *p*-values of TWAS were replicable in large samples. Collectively, our results provide a powerful new resource for integrating gr-expression with population genetics of brain organization and disease.

## Abstract 2

Integrating neuroimaging and molecular brain data across people and brain regions and states can help to understand human brain individuality. This study presents a resource that uses computational genomics to estimate genetically regulated components of gene expression and infer associations of this expression with neuroimaging and clinical phenotypes.

## Introduction

Much of human neuroscience seeks to understand the biological basis of individual variation in brain organization [1–6]. Studies have shown that this variation is stable over time [7,8], predicts function or behavior [9,10], and can act as a fingerprint of healthy [11,12] and diseased [13,14] brain states. They have also shown that much of this variation is strongly heritable and therefore genetically encoded [15–18]. Separately, complementary studies have shown the presence of correlated variation in gene expression and neural organization across brain regions [19–27]. Collectively, this literature motivates the need for integrative analyses of brain individuality across people and brain regions.

Such integrative analyses ultimately require data on genomes, brain-wide gene expression, as well as neuroimaging and clinical phenotypes in the same human populations. Correspondingly, such analyses are hampered, at present, by the lack of these multifaceted data. Instead, the genetic basis of individual variation in neuroimaging phenotypes is primarily investigated with genome-wide association studies (GWAS) [16–18,28–32]. Prominent examples of these studies have used data from the ENIGMA Consortium [33,34], the UK Biobank [35–37], and the ABCD Project [38]. These studies have linked variation in phenotypes to single-nucleotide polymorphisms (SNPs), variants of DNA base pairs at specific positions in the genome. Strengths of these studies include the ability to scan whole genomes and to directly discover nucleotide-level underpinnings of neuroimaging phenotypes. Limitations of these studies include the inability to disambiguate correlated association patterns of adjacent SNPs (known in genetics as linkage disequilibrium) and, more generally, to identify biological mechanisms of variation in neuroimaging phenotypes. They also include the need to test millions of associations (1 test for each pair of SNP and phenotype) and the consequent burden on statistical power necessitated by stringent correction for these many tests. In practice, robust GWAS for many complex phenotypes, such as height or blood pressure, can require samples from millions of people [39–41]. The costs of imaging the brain, however, make it impossible to acquire samples of this size in neuroimaging research [42]. Collectively, these limitations have left gaps in existing analyses of human brain individuality.

Here, we help to bridge these gaps by estimating genetically regulated gene expression, or gr-expression, across cortical and subcortical brain regions. Gene expression is regulated by multiple genetic and environmental factors. Our estimation focuses on one of these factors, genetically encoded elements that are close to the gene along the linear genome (*cis*-genetic regulation) [43]. We do not consider other factors, including genetically encoded elements far from the gene (*trans*-genetic regulation), as well as environmental factors. The genetics literature includes a variety of methods for estimating regional gr-expression from genetic data [44,45]. Our study uses Joint-Tissue Imputation, a state-of-the-art method that trains linear regression models of gr-expression on directly measured gene expression from postmortem samples [43].

We used this estimated gr-expression to perform transcriptome-wide association studies or TWAS. We specifically associated Joint-Tissue estimates of gr-expression with neuroimaging phenotypes and brain-related clinical phenotypes. TWAS follow the same methodology as GWAS, except that they link variation of neuroimaging phenotypes to regionally specific gr-expression of genes, rather than to regionally agnostic variation of SNPs. TWAS have several advantages over GWAS: they integrate signals across multiple SNPs, provide interpretable results at the level of genes, are less susceptible to linkage disequilibrium, and require many fewer statistical tests. However, TWAS are also limited to genes with available estimates of regional gr-expression and, like GWAS, are ultimately association studies that cannot alone establish causal effects of genes on phenotypes[46].

TWAS are common in the wider genomics literature [44–48] but, despite their advantages, are rare in neuroimaging genomics. We hypothesize that one major reason for their lack of adoption lies in the relatively theoretical nature of their appeal to neuroimaging researchers. First, the indirect nature of estimated gr-expression can make it difficult to relate this quantity to directly assayed gene expression of regional transcriptomic studies. Second, the similarly indirect nature of TWAS can make it difficult to ascertain the practical advantages of these studies relative to the more established GWAS. For example, the few existing TWAS of neuroimaging phenotypes in the literature [49–52] have not benchmarked these analyses against GWAS. Third, and related to these limitations, the field lacks integrated resources that link associations of regional estimates of gr-expression and SNPs on the one hand, to neuroimaging and clinical phenotypes on the other hand.

We propose that overcoming these limitations can help facilitate the adoption of TWAS in neuroimaging genomics. Here, we help to do so by using estimated gr-expression to integrate large-scale genomic, transcriptomic, neuroimaging, and clinical data sets. First, we showed that patterns of estimated gr-expression recapitulate brain regional identities and inter-regional correlation structure of directly assayed gene expression. Second, we used these estimates to perform TWAS of gr-expression and gray-matter volumes in the UK Biobank data set [35–37]. We directly benchmarked these TWAS against GWAS to show broad similarities but also important differences in the interpretability and statistical power of these approaches. Third, we integrated our results with an independent TWAS of brain-related clinical phenotypes from BioVU, the Vanderbilt Biobank [53]. This integration linked SNPs and genes to neuroimaging and clinical phenotypes through associations with estimated gr-expression. Fourth, we built polygenic models of gr-expression to discover associations of gr-expression with neuroimaging phenotypes in the Human Connectome Project (HCP) [54], a small neuroimaging-genomic data set with high-quality functional imaging data. Finally, we showed that estimates of gr-expression were replicable in an independent data set. We also showed that magnitudes of TWAS were generally replicable while *p*-values of TWAS were replicable in large samples of the UK Biobank. We developed a browser-based application for interactive exploration of our multifaceted association results. Collectively, our analyses help to facilitate the adoption of TWAS in neuroimaging genomics.

## Results

### Estimation of genetically regulated gene expression across brain regions at biobank scale

We used Joint-Tissue Imputation [43], a recently developed state-of-the-art method from computational genomics, to estimate the genetically regulated expression of 18,647 genes across 10 cortical and subcortical brain regions for 45,549 people from the UK Biobank (64 ± 7.7 years old, 52% female) and 657 people in the HCP (29 ± 3.6 years old, 52% female).

**Fig 1:**
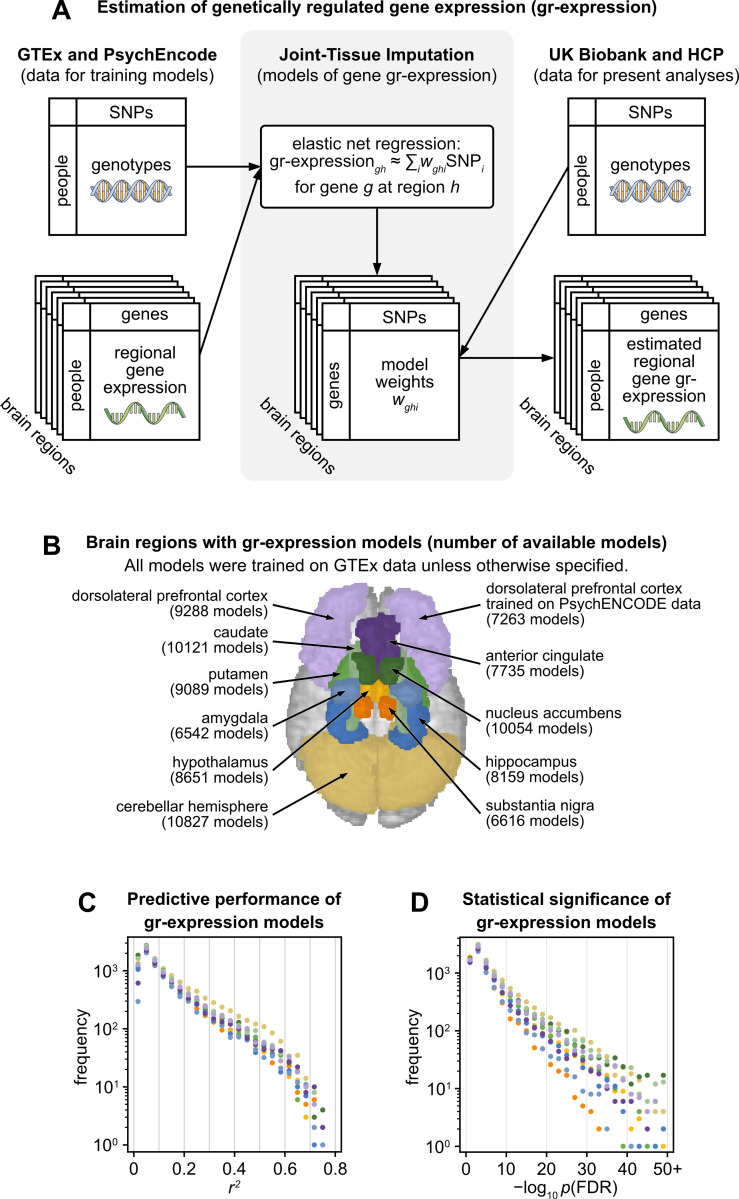
Estimation of genetically regulated gene expression from genetic data. **(A)** Pipeline for estimation of gr-expression with Joint-Tissue Imputation. Left: Joint-Tissue Imputation models are trained on genetic sequences and directly assayed gene expression from postmortem brain samples in the GTEx and PsychEncode projects. Center: The models are trained to estimate gr-expression as a weighted sum of SNPs that are close to the gene of interest along the linear genome. The estimation includes elastic-net regularization because the number of these SNPs typically exceeds the number of samples in the training data. Right: The trained models were used to estimate gr-expression from genetic sequences of neuroimaging-genomic samples in the UK Biobank and the HCP. **(B)** An illustration of the 10 cortical and subcortical regions with available models of gr-expression. Numbers in parentheses refer to all models that passed baseline performance thresholds for the prediction of observed gene expression on held-out data (*r*^2^ > 0.01 and *p*_FDR_ < 0.05). **(C, D)** Predictive performance of gr-expression models on held-out data from the GTEx data set. **(C)** Histograms of *r*^2^, the variance of directly assayed gene expression explained by estimated gr-expression. **(D)** Histograms of *p*-values (−log_10_ *p*_FDR_) on these *r*^2^ values. Regions are colored as in panel **B**. FDR, false discovery rate; GTEx, Genotype-Tissue Expression Project; HCP, Human Connectome Project; SNP, single-nucleotide polymorphism.

Joint-Tissue Imputation models estimate genetically regulated gene expression (gr-expression) as a weighted linear combination of SNPs that are close to the gene of interest along the linear genome. These models learn weights for each tissue–gene pair by training on genetic sequences and directly measured gene expression from postmortem samples (**Fig 1A**). Joint-Tissue Imputation leverages shared patterns of genetic regulation across brain regions to improve the estimation of gr-expression in individual regions. In this way, this method extends and generalizes PrediXcan, a pioneering estimation method that models gr-expression by training models only on expression data from the brain region of interest [55].

In our study, we used Joint-Tissue Imputation models that were previously trained on whole-genome sequences and gene-expression data from 838 brain samples in the Genotype-Tissue Expression Project (GTEx) [56]. The samples comprise 10 cortical and subcortical regions (**Fig 1B**). To test the replicability of our analyses, we additionally used the same models trained on sequencing and expression data from 415 independent samples of the dorsolateral prefrontal cortex (DLPFC) in the PsychENCODE Project [57]. Collectively, we considered 94,345 Joint-Tissue Imputation models, or all performant brain-regional models currently available in the literature.

Joint-Tissue Imputation models have been extensively validated in previous work [44–48]. This validation included quantifying the relationship of gr-expression to directly assayed expression. In this study, we adopted all models of gr-expression that passed baseline performance thresholds for the prediction of observed gene expression on held-out data (*r*^2^ > 0.01 and *p*_FDR_ < 0.05). In practice, the predictive performance of gr-expression models spanned a wide range (**Fig 1C and 1D**). Low predictive performance does not necessarily mean that the models are inaccurate because the genetic regulation of gene expression—the upper bound on predictive performance—varies considerably for individual genes. Moreover, relatively low associations between gr-expression and assayed expression are more than offset by gains in statistical power of transcriptome-wide association analyses, as we describe below.

### Genetically regulated gene expression recapitulates the organization of directly assayed gene expression

We began by testing the extent to which gr-expression recapitulated existing knowledge of genetic-ancestry relationships, brain-regional identities, as well as inter-regional correlations of directly assayed gene expression.

**Fig 2:**
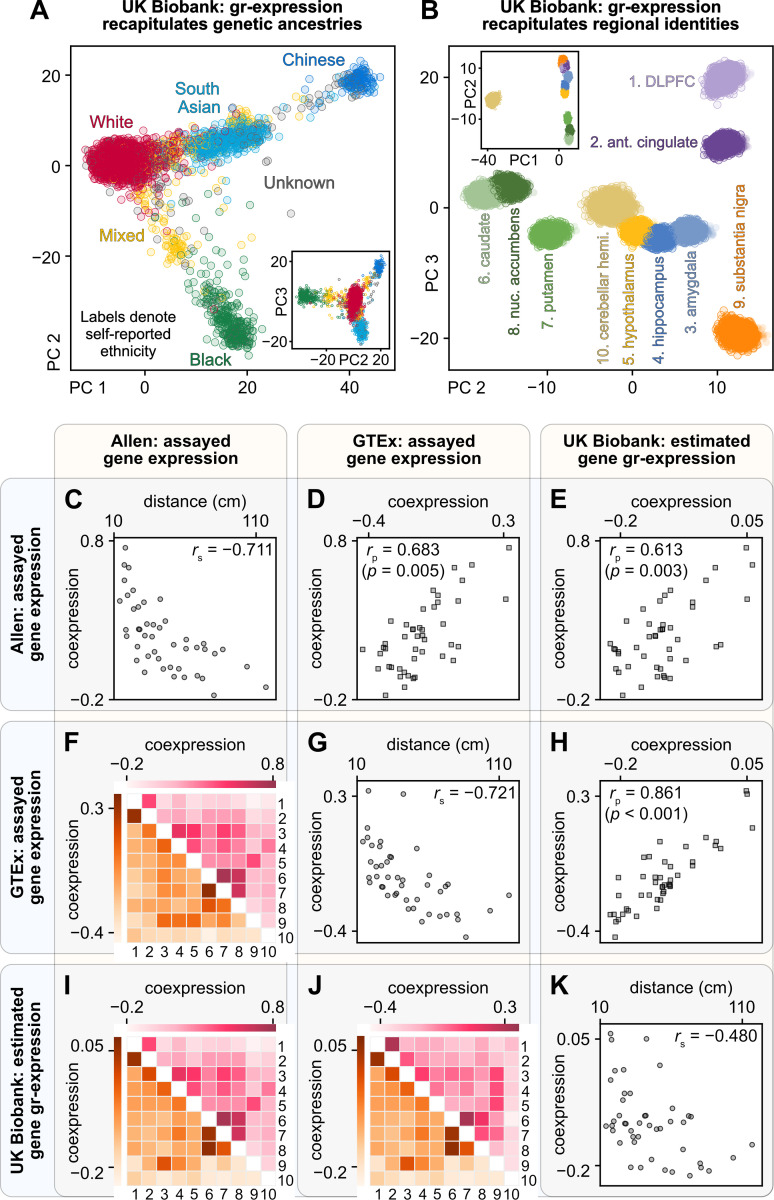
Genetic ancestry, regional identity, and inter-regional organization of estimated gr-expression. **(A, B)** Principal component embeddings of estimated gr-expression from the ethnically diverse sample of the UK-Biobank cohort (**S1 Table**). **(A)** An embedding of brain-wide gr-expression: scatter plots of principal components of the *people* × [*brain-wide gr-expression*] matrix, where *people* denote people from the UK-Biobank sample and *brain-wide gr-expression* denotes brain-wide estimates of gr-expression for all genes that had Joint-Tissue Imputation models for each of the 10 regions. **(B)** An embedding of regional gr-expression: scatter plots of principal components of the *regions* × [*regional gr-expression*] matrix where *regions* denote the 10 regions of people from the UK-Biobank sample and *regional gr-expression* denotes regional estimates of gr-expression for all genes that had Joint-Tissue Imputation models for each of these regions. **(C–K)** A 3 × 3 matrix of plots of inter-regional coexpression: correlations between directly assayed expression and estimated gr-expression. The first row and column show results on directly assayed gene expression data from the Allen Human Brain Atlas. The second row and column show results on directly assayed gene expression data from the GTEx project. The third row and column show results on estimated gr-expression from the ethnically diverse sample of the UK-Biobank sample. **(C, G, K)** Associations between inter-regional coexpression and Euclidean distance in each data set. **(D, E, H)** Associations between inter-regional coexpression across data sets. *P*-values denote the probability of obtaining coexpression of at least equal magnitude in data with preserved correlation coefficients between coexpression and Euclidean distance (estimated from 10,000 random samples). **(F, I, J)** Heatmaps of inter-regional coexpression, averaged across people in each data set (regional numbers follow numbers in panel **B**). DLPFC, dorsolateral prefrontal cortex; GTEx, Genotype-Tissue Expression Project.

First, we tested if gr-expression patterns reflected known genetic-ancestry relationships from the ethnically diverse sample of the UK-Biobank cohort (**Methods**, **S1 Table**). Genetic ancestry denotes genetic commonalities within groups of people but does not necessarily reflect genealogical ancestry (family lines) or self-reported ethnicity. We followed standard practice to estimate genetic ancestry using principal component analysis of gene data. We specifically used principal component analysis to generate low-dimensional embeddings of brain-wide gr-expression from each person (using the *people* × [*brain-wide gr-expression*] matrix). As expected, this analysis partitioned people into clusters of African, Asian, and European populations with gradients between these clusters reflecting known patterns of genetic admixture (**Fig 2A**). This embedding reflects patterns of genetic ancestry that are known and were previously described in analyses of genetic-sequence data [58].

Second, we tested if gr-expression patterns reflected regional brain identities across people in the same sample. For this analysis, we generated principal component embeddings of individual region-specific gr-expression (using the *regions* × [*regional gr-expression*] matrix). This analysis partitioned gr-expression into well-delineated regional clusters and revealed anatomically interpretable groups of cortical, limbic, and basal ganglionic clusters (**Fig 2B**). Collectively, these results show that gr-expression simultaneously reflects genetic-ancestry identities across people and brain-regional identities within people. They imply, specifically, that associations of gr-expression, or TWAS, can capture variation across people, similarly to GWAS, as well as variation across regions, similarly to regional transcriptomic studies.

Third, we compared inter-regional correlations of estimated gr-expression to inter-regional correlations of directly assayed expression data from the Allen Human Brain Atlas and the GTEx Project. Recent studies have shown that inter-regional coexpression exponentially decays as a function of inter-regional distance [59,60]. We reproduced these relationships by showing strong inverse nonlinear relationships between inter-regional coexpression in the Allen and GTEx data and Euclidean distance: Allen versus distance *r*_spearman_ = −0.711 and GTEx versus distance *r*_spearman_ = −0.721 (**Fig 2C and 2G**). We found a similar, albeit weaker, relationship in the estimated gr-coexpression data: UK Biobank versus distance *r*_spearman_ = −0.480 (**Fig 2K**). More directly, we found strong linear relationships between the inter-regional coexpression in the Allen and GTEx data: Allen versus GTEx *r*_pearson_ = 0.683 (**Fig 2D**). We found similar relationships between estimated and directly assayed inter-regional coexpression: UKB versus Allen *r*_pearson_ = 0.613 and UKB versus GTEx *r*_pearson_ = 0.861 (**Fig 2E and 2H**). Heatmaps of all coexpression patterns reflected associations between cortical, basal ganglionic, and other subcortical systems (**Fig 2F, 2I and 2J**). Finally, we showed that the relationship of coexpression with distance was not sufficient to explain these similarities of coexpression (*p* ≤ 0.005 for all tests).

Collectively, these results provide multifaceted support for the biological validity, anatomical interpretability, and practical utility of estimated gr-expression. In this way, they establish a foundation for the use of gr-expression in neuroimaging TWAS.

### TWAS link genetically regulated gene expression with regional gray-matter volumes

**Fig 3:**
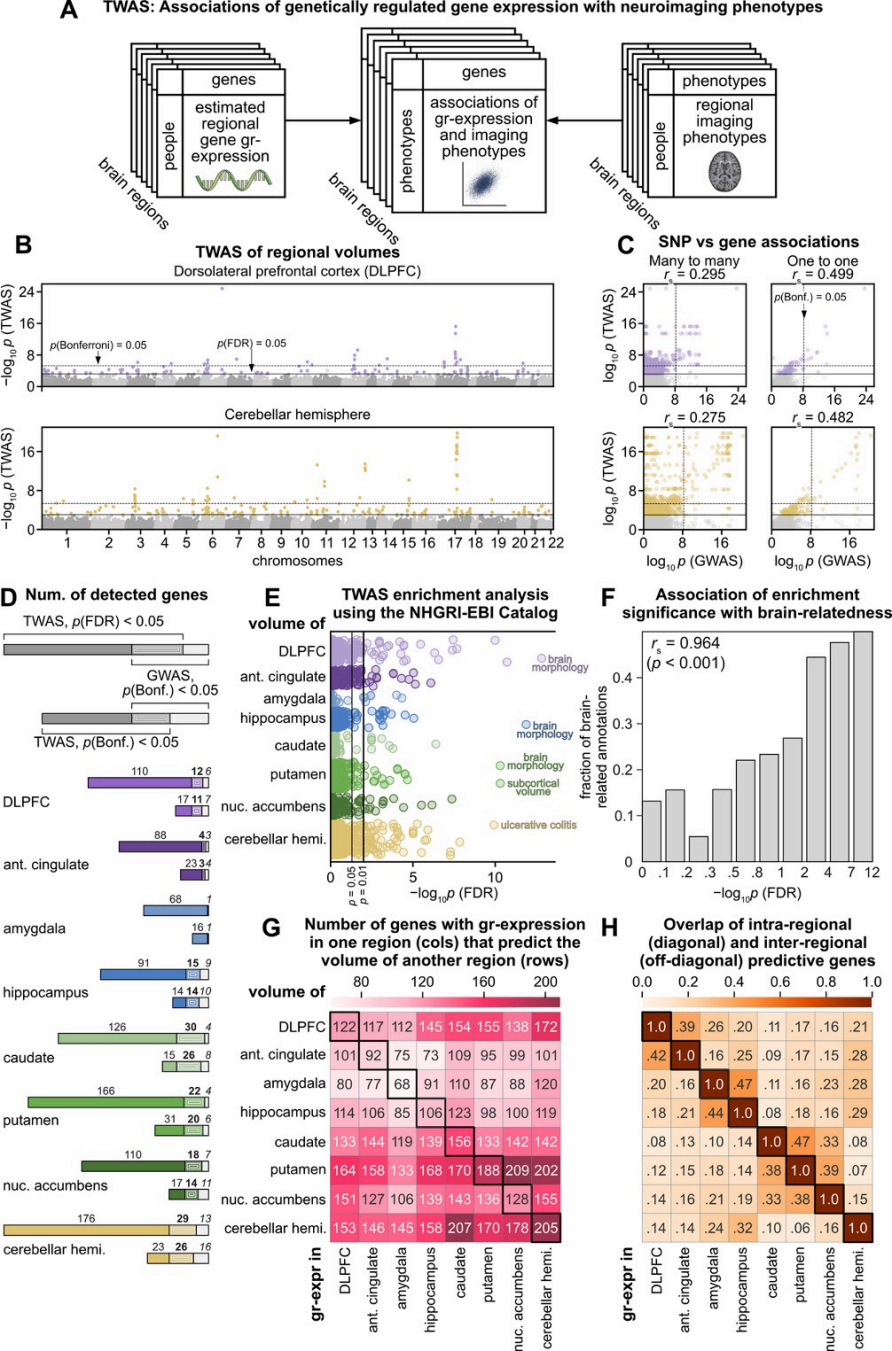
Variation of regional gr-expression and regional volumes across people. **(A)** A pipeline for transcriptome-wide association studies, or TWAS, of neuroimaging phenotypes. The inputs to TWAS comprise values of regional gr-expression (left) and regional phenotypes (right), estimated in the same people. The outputs are associations between the individual variation of regionally specific gr-expression and neuroimaging phenotypes across people (center). **(B)** Within-regional associations of gr-expression and gray-matter volumes for 2 representative regions. Each point denotes an association between the individual variation of gr-expression and volume in the same region. The horizontal axis shows the chromosome location of individual genes. The vertical axis shows the *p-*values (–log_10_ *p*) of associations. Solid-color points represent associations that pass the thresholds of *p*_FDR_ = 0.05 or *p*_Bonferroni_ = 0.05 (horizontal lines). Source data can be found in S2 Table. **(C)** Associations between SNP-based GWAS and gene-based TWAS for 2 representative regions. Left: Scatter plots of *p-*values (–log_10_ *p*) for associations of all genes and SNPs. These plots preserve all genes and SNPs but lack the one-to-one relationship between genes and SNPs. Right: Corresponding scatter plots for the best-performing genes and SNPs. Each gene in TWAS matches with its best-performing SNP in GWAS. Similarly, each SNP in GWAS matches with its best-performing gene in TWAS. These plots show one-to-one relationships but exclude many genes and SNPs. **(D)** Numbers of associations (*p*_FDR_ < 0.05 or *p*_Bonferroni_ < 0.05) detected with TWAS and GWAS. Solid colors denote numbers of associations detected with TWAS alone. Beige colors denote number of genes detected with GWAS alone. Stripe patterns denote numbers of genes detected with both TWAS and GWAS. The top bar for each region adopts an FDR correction for TWAS associations (*p*_FDR_ < 0.05), while the bottom bar adopts a stricter Bonferroni correction (*p*_Bonferroni_ < 0.05). **(E, F)** Enrichment analyses of TWAS for biological annotations in the NHGRI-EBI GWAS Catalog. **(E)** Enrichment for biological annotations of genes whose gr-expression predicted regional volumes (*p*_FDR_ < 0.05). Each point represents a biological annotation associated with at least 1 gene. The horizontal axis shows the *p*-values (–log_10_ *p*_FDR_) of individual annotations. Source data can be found in S3 Table. **(F)** Relationship between *p*-values and brain-relatedness of biological annotations. The horizontal axis shows bins of *p*-values (–log_10_ *p*_FDR_). The vertical axis shows the fraction of brain-related annotations within each bin. The *p*-value on the correlation coefficient was computed by permuting the annotations (estimated from 10,000 random samples). **(G, H)** Heatmaps of inter-regional TWAS between gr-expression and regional volumes. **(G)** Absolute numbers of associations. Numbers of genes whose gr-expression in 1 region (columns) predicted (*p*_FDR_ < 0.05) the volume of another region (rows). Source data can be found in S4 Table. **(H)** Overlap coefficients. Number of genes that were common to both intra-regional and inter-regional associations in **G**, normalized by the size of the smaller of the intra- and inter-regional gene sets. FDR, false discovery rate; GWAS, genome-wide association studies; SNP, single-nucleotide polymorphism; TWAS, transcriptome-wide association studies.

We hypothesized that the integration of multiple SNPs into models of regional gr-expression would allow us to detect novel and neurobiologically meaningful associations. To test this hypothesis, we performed TWAS to identify associations between individual variation of regional gr-expression and gray-matter volumes (**Fig 3A**). Gray-matter volumes are heritable phenotypes that have been linked to many genetic variants in previous GWAS [16,17,31]. We focused our association studies on 8 regions with available FreeSurfer [61] segmentations and therefore excluded substantia nigra and hypothalamus from subsequent analyses (see **Methods** for regional definitions).

Our first TWAS inferred associations between gray-matter volumes and gr-expression of the same regions. To minimize the confounders of genetic ancestry, we restricted our analyses to the “White British” sample of the UK-Biobank cohort (**S1 Table**) [37]. We therefore performed TWAS on 39,565 people (52.2% female, 64.3 ± 7.7 years old), with covariates of genetic ancestry, sex, and age (**Methods**).

We identified 1,065 associations (of 778 unique genes) between gr-expression and the volumes of 8 brain regions (*p*_FDR_ < 0.05, **Fig 3B and S1 and S2 Tables**). The number of regional associations varied from 68 genes in the amygdala to 205 genes in the cerebellar hemisphere. Many genes that were found in this analysis, including *CRHR1*, *ARL17A*, *NSF*, and *OGFOD2*, have been implicated in previous GWAS of regional brain volumes, and have also been linked to brain disorders, including epilepsy, schizophrenia, and brain cancer [62–64].

### TWAS reinforce GWAS associations and discover novel associations

To directly show the methodological advantages of gene-based TWAS, we directly compared these studies to SNP-based GWAS. We made this comparison in 3 complementary ways.

*Direct relationship to GWAS*. First, we performed a GWAS on the same sample and compared our TWAS associations for individual genes to GWAS results for the SNPs that formed part of corresponding models of gr-expression. These comparisons were dominated by many-to-many relationships between genes and SNPs, because several SNPs typically predict the gr-expression of a single gene, and similarly, a single SNP can help predict the gr-expression of several genes. The correlations between GWAS and TWAS *p*-values were moderate but statistically significant (0.275 ≤ *r*_spearman_ ≤ 0.373, *p* < 0.001 for all regions, **Fig 3C left**, **S1 Fig**). To focus on the strongest TWAS and GWAS signals, we filtered these data in a way that retained the lowest *p*-value SNP for each gene and, simultaneously, the lowest *p*-value gene for each SNP. This process resulted in much stronger and strictly one-to-one relationships (0.479 ≤ *r*_spearman_ ≤ 0.583, *p* < 0.001 for all regions, **Fig 3C right** and **S1 Fig**). Collectively, these results show that gene-based TWAS associations are related to, but also distinct from, SNP-based GWAS associations.

*Statistical power*. Second, we investigated the nature of these differences by contrasting the number of associations detected by TWAS and GWAS. The high multiple-testing burden of GWAS typically requires strict genome-wide Bonferroni corrections. By contrast, the relatively smaller number of statistical tests in TWAS results in a lower multiple testing burden, and the expected polygenic associations of many phenotypes make it common to adopt less strict false discovery rate (FDR) corrections as an alternative to Bonferroni [48]. In our analyses, TWAS under both corrections identified many more genes than the corresponding GWAS (**Fig 3D**). Specifically, under FDR correction, TWAS detected associations of 673 unique genes (*p*_FDR_ < 0.05) that lacked GWAS associations of corresponding SNPs (*p*_Bonferroni_ < 0.05). Many of these genes have been previously linked to brain-related disorders, including Alzheimer’s disease (*WDR12*, *AGFG2*, and *CDK5RAP3*), schizophrenia (*SRA1*, *WDR55*, *CORO7*, *DDAH2*, *PCDHA8*), autism spectrum disorder (*MAPK3*, *PCDHA13*), and major depressive disorder (*ZMAT2* and *ITIH4*) [65–74]. Separately, under Bonferroni correction, TWAS detected associations of 110 unique genes (*p*_Bonferroni_ < 0.05) that lacked GWAS associations of corresponding SNPs (*p*_Bonferroni_ < 0.05). These results show that TWAS discovers associations of many genes that are undetected with GWAS.

*Neurobiological interpretability*. Third, to interpret the function of discovered genes more systematically, we tested the enrichment of our TWAS results using the NHGRI-EBI GWAS Catalog, a catalog of gene annotations curated from all human GWAS in the current literature [75]. We discovered 276 enriched biological annotations at *p*_FDR_ < 0.05 (**Fig 3E and S3 Table**) and found that brain-related annotations were much more likely to be enriched than other annotations in the catalog (*p* < 0.001). Moreover, in addition to the overall enrichment for brain-related annotations, we found a strong positive correlation between the *p*-values of the enrichment and the fraction of discovered brain-related annotations (*r*_spearman_ = 0.964, *p* < 0.001, **Fig 3F**). In other words, we found that the most enriched gene annotations were primarily brain related. **S2 Fig** shows that these enrichments were replicable with a Bonferroni correction on TWAS associations. Collectively, these results show the neurobiological relevance of our discoveries.

### TWAS discover associations of genetically regulated gene expression in one brain region with gray-matter volumes of other regions

Separately, we built on our region-specific TWAS findings to test for associations between gr-expression in one brain region and gray-matter volumes of other regions. Such associations are undefined for SNPs (because all cells share the same genome), but are interpretable for gr-expression (because of known inter-regional similarities in gene expression and organization [15,20,23,25]). In practical terms, these analyses also help to discover associations of regional volumes with genes for which these regions currently lack models of gr-expression (**Fig 1B**).

Inter-regional TWAS discovered between 73 and 209 (median 133) associations (*p*_FDR_ < 0.05) of gr-expression in one region with the volume of another region (**Fig 3G and S4 Table**). gr-Expression in the amygdala and anterior cingulate had the largest number of such associations (**Fig 3G**, columns) relative to the total available number of gr-expression models in each region (**Fig 1B**). For example, the gr-expression of *FOXO3* in the anterior cingulate predicted the volumes of all 8 regions. This gene has been strongly linked to healthy aging in diverse human populations [76–78]. By contrast, the volume of putamen was predicted by the largest number of genes from other regions (**Fig 3G**, rows). Several of these genes—including *MYLK2*, *KTN1*, *DCC*, *BCL2L1*, *TPX2*, and *HELZ*—were associated with putamen volume in previous studies [16,79–84]. In particular, in our study, the gr-expression of *MYLK2* and *KTN1* predicted putamen volume in all regions that had gr-expression models of these genes (in 8 and 4 regions, respectively). In other cases, gr-expression of some genes in many regions predicted volumes of many other regions. For example, the gr-expression of *LRRC37A2* in all 8 regions predicted volumes of all regions except putamen and caudate. Similarly, gr-expression of *MAPT* in the cerebellar hemisphere predicted all volumes except putamen and caudate. Both *LRRC37A2* and *MAPT* have been linked to Parkinson’s disease, and *MAPT* encodes for tau and has been well studied in the Alzheimer’s disease literature [50,85,86].

We finally quantified the overlap between intra-regional and inter-regional associations. A heatmap of overlap coefficients of these associations formed 3 anatomically distinct groupings of cortical, basal ganglionic, and limbic regions (**Fig 3H**). These groupings show that the volumes of anatomically similar regions are more likely to share gene associations or, alternatively, that genes from one region are associated with volumes of anatomically similar regions. **S2 Fig** shows that these groupings were replicable with a Bonferroni correction on TWAS associations.

Collectively, these results suggest a strong relationship between gr-expression profiles of anatomically similar brain regions and, more generally, show the utility of inter-regional TWAS of neuroimaging phenotypes.

### Genetically regulated gene expression links regional volumes with clinical phenotypes

**Fig 4:**
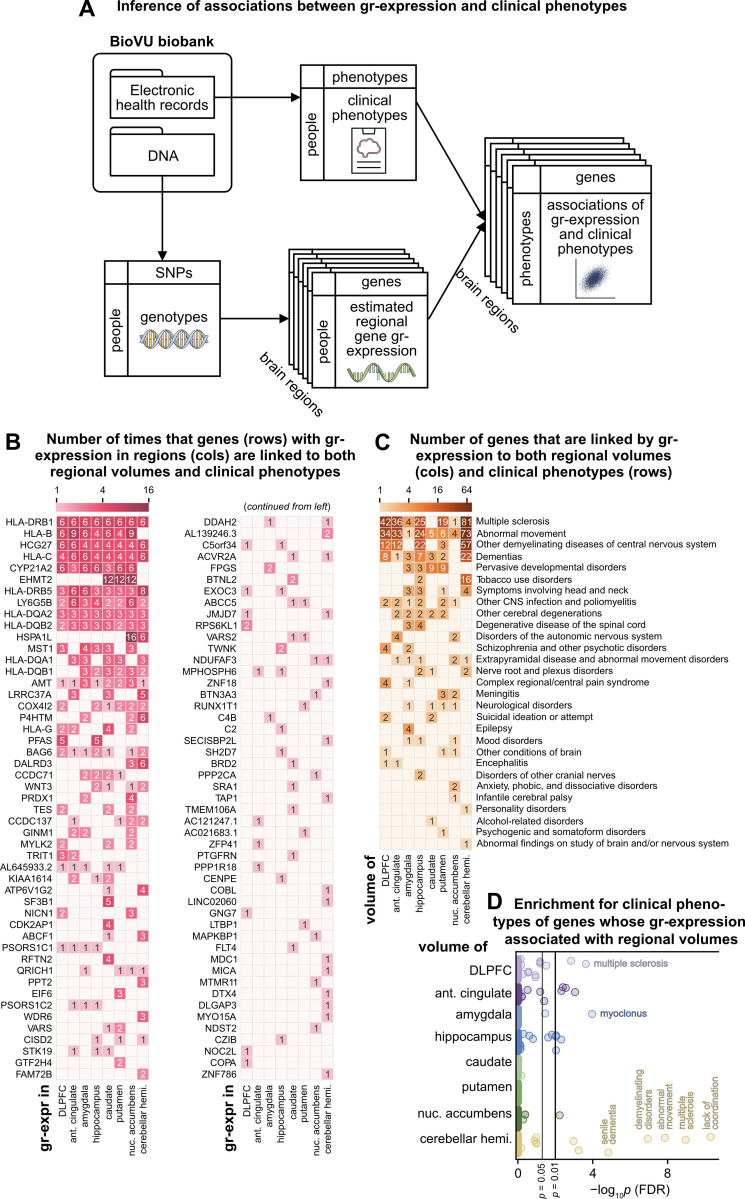
Association of gr-expression with both neuroimaging and clinical phenotypes. **(A)** Pipeline for BioVU TWAS: transcriptome-wide association studies of regional gr-expression and clinical phenotypes from the BioVU Biobank. Top left: Inputs to TWAS comprise electronic health records and DNA samples of the same people. Top center: Clinical phenotypes are extracted from ICD-9 codes present in electronic health records. Bottom left and center: Regional gr-expression is estimated from DNA samples of the same people. Right: Clinical phenotypes and regional gr-expression are combined in the BioVU TWAS. **(B)** Heatmap showing the number of times by which genes (rows) with regional gr-expression (columns) were linked to both regional volumes and clinical phenotypes. Each count denotes a regional gr-expression that was associated (*p*_FDR_ < 0.05) with both a regional volume in the UK Biobank TWAS and with a brain-related clinical phenotype in the BioVU TWAS. **(C)** Heatmap showing the number of genes with regional gr-expression that linked regional volumes (columns) with clinical phenotypes (rows). Each count denotes a regional gr-expression that was associated (*p*_FDR_ < 0.05) with both a regional volume in the UK Biobank TWAS and with a brain-related clinical phenotype in the BioVU TWAS. **(D)** Enrichment of clinical phenotypes for genes whose gr-expression predicted (*p*_FDR_ < 0.05) regional volumes (rows) in the UK Biobank TWAS. Each point represents a brain-related clinical phenotype associated with at least 1 gene. The horizontal axis shows the *p*-values (–log_10_ *p*_FDR_) of individual phenotypes. Source data can be found in **S5 Table**. FDR, false discovery rate; TWAS, transcriptome-wide association studies.

We next moved beyond literature-based annotations to test whether gr-expression associations can link regional volumes with clinical phenotypes. To achieve this, we integrated our results with a separate TWAS on a sample of 70,439 people in BioVU, a biobank that contains DNA samples and de-identified electronic health records for patients at Vanderbilt University Medical Center [53,87,88]. Clinical phenotypes derived from electronic health records in BioVU were represented by phenotype codes extracted from International Classification of Diseases (ICD-9) billing codes. The BioVU TWAS used the same Joint-Tissue Imputation models to estimate gr-expression and to discover clinical associations (**Fig 4A**). In what follows, we filtered this clinical TWAS to focus on 156 brain-related clinical phenotypes. We then compared associations of regional gr-expression with these phenotypes to associations in our inter-regional neuroimaging TWAS.

We identified 98 genes whose gr-expression in a specific region associated (*p*_FDR_ < 0.05) with both volumes in the UK Biobank TWAS and with brain-related clinical phenotypes in the BioVU TWAS (**Fig 4B**). There were 22 genes in this set whose gr-expression in 4 or more regions linked volumes and clinical phenotypes. In previous GWAS and clinical studies, these genes have been associated with neurogenesis (*WNT3*) [89,90], neurodevelopmental delays (*QRICH1*) [91,92], addiction (*HCG27*) [93], depression (*CCDC71*, *CYP21A2*) [94,95], and other brain-related disorders [96,97].

BioVU clinical phenotypes that shared associations of gr-expression with regional volumes included a variety of nervous system symptoms and disorders including, most prominently, demyelinating diseases, motor-related symptoms, and dementia (**Fig 4C**). Several *HLA* genes that play a major role in the immune response (including *HLA-B/C*, *HLA-DRB1*, and *HLA-DRB5*) were associated with 2 or more regional volumes and simultaneously with demyelinating diseases, including multiple sclerosis, a prominent immune-mediated disorder [98]. In addition, genes in the *HLA-DR* and *HLA-DQ* families were associated with volumes of the cerebellar hemisphere and hippocampus in the UK Biobank and simultaneously with the *abnormal movement* phenotype in the BioVU TWAS. These associations represent candidate causal mechanisms for linking these genes with Parkinson’s disease and other movement disorders [99–102]. Genes *C4B*, *MST1*, and *LRRC37A* showed similar patterns of associations, in this way supporting and expanding previous links to motor disorders [86,103–105].

Separately, we identified 9 brain-related clinical phenotypes that were enriched (*p*_FDR_ < 0.05) for genes whose gr-expression predicted regional volumes (**Fig 4D and S5 Table**). Most of these phenotypes were enriched for genes that predicted multiple regional volumes. For example, *myoclonus* was enriched for genes that predicted volumes of 6 regions, while *multiple sclerosis* and *lack of coordination* were enriched for genes that predicted volumes of 4 regions. Further, *senile dementia* was enriched for genes that predicted hippocampal and cerebellar volumes, while *speech disturbances* was enriched for genes that predicted anterior cingulate volume. The majority of motor-related clinical phenotypes were enriched for genes that predicted volumes of the cerebellum, a well-known center of motor control. **S3 Fig** shows that our association and enrichment analyses were replicable with a Bonferroni correction on TWAS associations.

Overall, these results show that associations of gr-expression with phenotypes at different biological scales can be combined to reveal genes that link regional volumes and clinical phenotypes. Despite differences in samples and phenotype modalities, we identified a large overlap in the 2 TWAS between associations with regional gr-expression. Furthermore, we found evidence in related literature that supports associations between regional volumes and an array of brain-related disorders. Collectively, these findings highlight the integrated relationships between gene expression and brain phenotypes and the implications of these relationships for the study of brain-related disorders.

### Polygenic models of genetically regulated gene expression detect associations in a small neuroimaging data set

**Fig 5:**
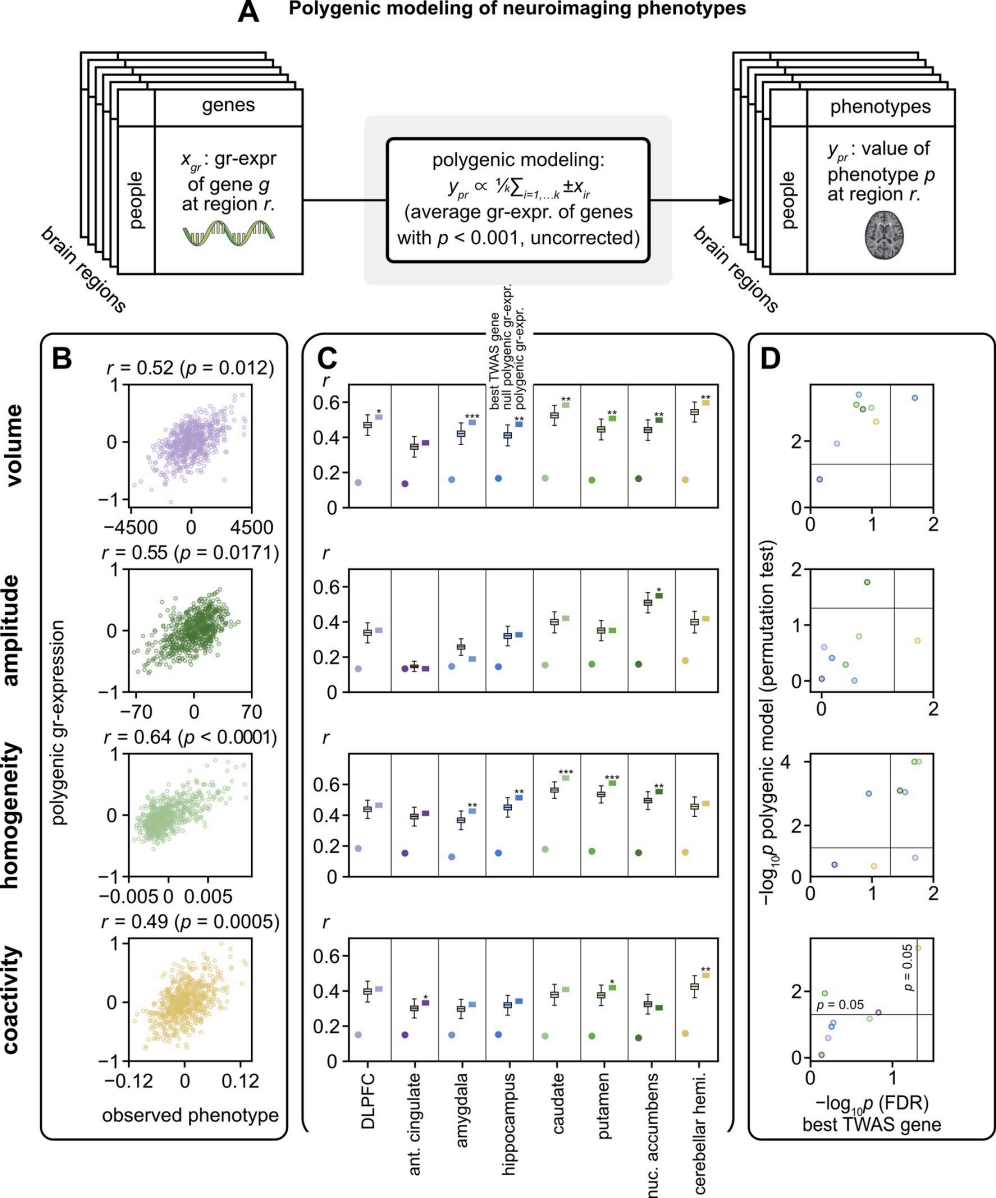
Associations of polygenic gr-expression with neuroimaging phenotypes. **(A)** A framework for polygenic modeling of regional phenotypes. Polygenic gr-expression was defined as the mean normalized gr-expression of genes that were nominally associated with phenotypes at *p* < 0.001, uncorrected. To make the mean well defined, the signs of gr-expressions with negative associations were reversed. Model performance was evaluated using permutation testing. **(B)** Representative scatter plots of neuroimaging phenotypes and polygenic gr-expression. Points represent individuals, and colors denote regions as labeled in **C. (C)** Pearson correlation coefficients between neuroimaging phenotypes and polygenic gr-expression (square points), polygenic gr-expression from permutation tests (box plots; *n* = 10,000), and best single-gene gr-expression from TWAS (round points). Stars represent *p*-values of polygenic associations with permutation testing (* *p* < 0.05, ** *p* < 0.005, *** *p* < 0.0005). Source data can be found in S1 Data. **(D)** Comparison of *p*-values (−log_10_ *p*_FDR_) from polygenic gr-expression associations and best single-gene TWAS. Colors denote regions, while lines denote *p* = 0.05. DLPFC, dorsolateral prefrontal cortex; FDR, false discovery rate; TWAS, transcriptome-wide association studies.

Recent studies have shown the potential of combining the gr-expression of multiple genes into polygenic models to improve the prediction of phenotypes (**Fig 5A**) [106–108]. Such polygenic models may be particularly relevant for highly polygenic phenotypes of brain anatomy and activity. They can also capture the polygenic nature of structural and functional MRI phenotypes and further reduce the number of statistical tests.

Here, we tested the power of such analyses using the HCP [54], a small but prominent neuroimaging genomic data set with high-quality functional MRI data. To minimize the confounders of genetic ancestry, we restricted our analysis of this data set to a sample of 657 non-twins of European genetic ancestry. Our analyses considered regional volume phenotypes, as well as a representative set of functional MRI phenotypes. The functional MRI phenotypes track properties of regional activity (*amplitude* of low-frequency fluctuations [109]), within-regional correlation (regional *homogeneity* [110]), and average inter-regional correlated activity (mean *coactivity* [111]). Specifically, *amplitude* reflects the power of low-frequency activity, homogeneity reflects the extent of intra-regional correlated activity, while coactivity complementarily reflects the extent of inter-regional correlated activity (**Methods**). These phenotypes provide insights into the organization of brain activity and have been extensively studied in neuroimaging genomics [26,27,112–114].

We first performed a single-gene TWAS on these phenotypes. The relatively small size of our sample, however, necessarily resulted in few associations that survived corrections for multiple comparisons. For example, and in contrast to the UK Biobank TWAS, most regional phenotypes in this analysis showed no associations at *p*_FDR_ < 0.05. Moreover, as expected, the strongest associations in this sample had much higher *p*-values (best *p*_FDR_ = 0.017) than the strongest associations in the UK Biobank TWAS (best *p*_FDR_ = 1.34 × 10^−21^).

We then estimated polygenic gr-expression as the mean normalized gr-expression of genes that had nominal TWAS associations with phenotypes (*p* < 0.001, uncorrected). We tested associations of polygenic gr-expression against null associations of equivalently estimated polygenic gr-expression on data with randomized (permuted) assignment of phenotypes to subjects.

Associations of polygenic gr-expression with phenotypes had mean ± standard deviation *r* = 0.434 ± 0.113 (**Figs 5B** and **S4**). For regional volume and homogeneity phenotypes, these associations tended to be higher than null associations (*p* < 0.05) and have lower *p*-values than single-gene associations (**Fig 5C and 5D and S1 Data**). By contrast, for amplitude and coactivity phenotypes, these associations did not tend to be higher than null associations and had similar *p*-values as single-gene associations (**Fig 5C and 5D and S1 Data**). Note also that polygenic gr-expression estimated from more selected genes tended to have higher associations in absolute terms and relative to the null associations (**S5 Fig**). Collectively, these analyses show that polygenic modeling can further improve the ability of TWAS to infer associations of groups of genes with complex phenotypes.

### Replicability of estimated genetically regulated gene expression and TWAS

We finally tested the replicability of our analyses in 3 complementary ways.

**Fig 6:**
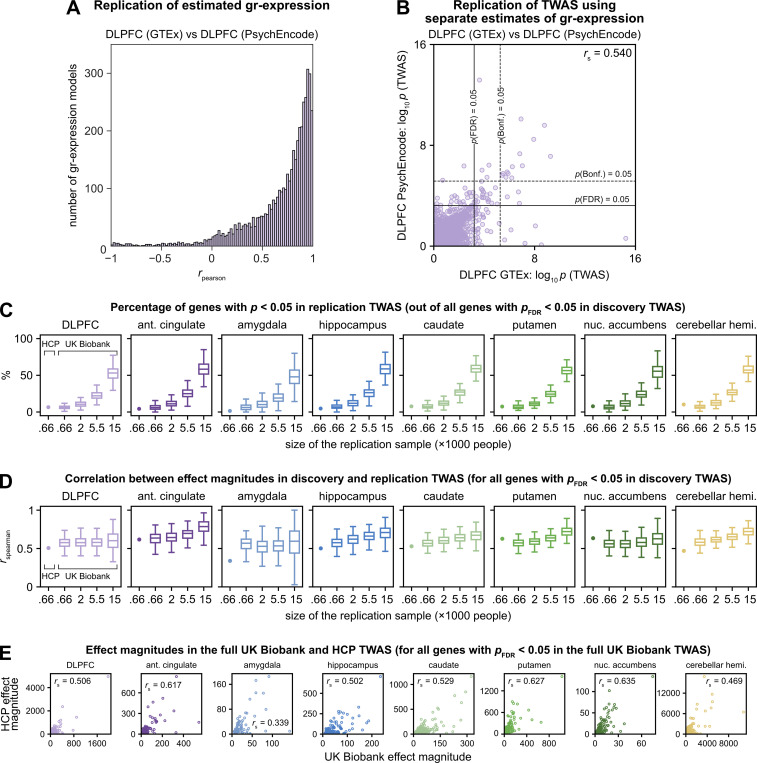
Replicability of estimated genetically regulated gene expression and TWAS. **(A, B)** Replication of estimated gr-expression trained on independent PsychEncode data. **(A)** Histogram of correlations between gr-expression of the DLPFC estimated with models trained on GTEx data and independent PsychEncode data. **(B)** Scatter plot of TWAS associations based on gr-expression of the DLPFC estimated with models trained on GTEx data and independent PsychEncode data. Each point denotes *p*-values of associations between estimated gr-expression and DLPFC gray-matter volumes in the white-British sample of the UK-Biobank cohort. **(C–E)** Replication of genes that passed *p*_FDR_ = 0.05 in discovery TWAS of gray-matter volumes. **(C)** Percentages of genes that were replicated at nominal *p* < 0.05 in replication TWAS. Source data can be found in S2 Data. **(D)** Correlations between effect magnitudes of genes in the replication and discovery TWAS. Dots denote analyses on the full UK Biobank (discovery) and HCP (replication) samples. Box plots denote analyses of discovery-replication splits of the white-British UK-Biobank sample, ordered from small to large replication samples. Each box plot was estimated from 300 random splits. **(E)** Scatter plots of effect magnitudes in the UK Biobank and HCP TWAS. Each point denotes effect magnitudes for a gene that showed *p*_FDR_ < 0.05 in the UK Biobank TWAS. DLPFC, dorsolateral prefrontal cortex; FDR, false discovery rate; GTEx, Genotype-Tissue Expression Project; HCP, Human Connectome Project; TWAS, transcriptome-wide association studies.

First, we tested the replicability of gr-expression models by comparing the estimated gr-expression of the DLPFC using models trained on 2 distinct postmortem samples: our main sample from GTEx and an independent replication sample from PsychEncode [43]. We found that models trained on the 2 samples had highly similar patterns of gr-expression (*r*_pearson_ of gr-expression: median 0.799, Q1–Q3 0.559–0.917, **Fig 6A**). Likewise, we found similar TWAS of these models with DLPFC volumes (*r*_spearman_ = 0.540, *p* < 0.001, **Figs 6B and S6**). These results suggest that our framework for estimating gr-expression is robust to the training data, at least for sufficiently large samples.

Second, we tested the replicability of association *p*-values and magnitudes in the UK Biobank using the independent HCP TWAS. As we saw above, the small HCP sample produced almost no associations at *p*_FDR_ < 0.05. Correspondingly, we found that a small percentage of associations with *p*_FDR_ < 0.05 in the UK Biobank were also present at the nominal threshold of *p* < 0.05 in the HCP TWAS (median 7.00%, Q1–Q3 4.53%–7.75%, **Fig 6C**). By contrast, the magnitudes of individual associations are strongly correlated with *p*-values (*r*_spearman_ between magnitudes and −log_10_*p*: median 0.783, Q1–Q3 0.773–0.786, **S6 Fig**) but, unlike *p*-values, are relatively independent of the sample size [115]. Correspondingly, we found consistently strong correlations between magnitudes of associations that passed *p*_FDR_ < 0.05 in the UK Biobank TWAS (*r*_spearman_: median 0.518, Q1–Q3 0.486–0.622, all *p* < 0.005, **Figs 6D, 6E and S6**).

Third, we repeated these analyses on TWAS of discovery and replication subsets generated from 1,200 random splits of the white-British UK-Biobank sample (**S2 Data**). These additional analyses showed that replication samples of the same size as our HCP sample (657 people) had similarly small percentages of replicable associations (median 6.60%, Q1–Q3 6.20%−6.82%) and that larger samples showed much higher percentages (**Fig 6C**). Likewise, these analyses showed that replication samples of the same size as our HCP sample had strong correlations between magnitudes of effects (*r*_spearman_: median 0.575, Q1–Q3 0.568–0.579; all *p* < 0.001) and that larger samples showed modestly increased correlations between magnitudes (**Figs 6D** and **S6**).

Collectively, these analyses suggest that the estimated gr-expression and magnitudes of TWAS associations were generally replicable, while the *p*-values of TWAS associations were replicable in large replication samples.

### Interactive application to facilitate adoption of TWAS in neuroimaging genomics

To increase the accessibility of our results, we created a browser-based application to explore our SNP-based and gene-based associations (https://github.com/nhunghoang/twas-webapp). The application allows users to compare neuroimaging GWAS with neuroimaging TWAS and with clinical TWAS and, in this way, links analyses of SNPs, genes, neuroimaging phenotypes, and clinical phenotypes. It also allows users to interactively explore associations and provides more direct gene-based interpretations of SNP-based results.

## Discussion

### Summary

We adopted state-of-the-art methods from computational genomics to estimate genetically regulated gene expression, or gr-expression, across 10 cortical and subcortical brain regions in more than 40,000 people. First, we showed that estimates of gr-expression across people and brain regions recapitulate the neurobiological organization of directly assayed gene expression. Second, we showed that TWAS based on estimated gr-expression aligned with, and extended, associations from corresponding GWAS. Third, we integrated these results with a set of independent associations between regional gr-expression and brain-related clinical phenotypes extracted from electronic health records. Fourth, we showed that polygenic models of gr-expression can further increase the statistical power of our approach. Finally, we showed that estimated gr-expression levels and the magnitudes of TWAS associations were generally replicable while the *p*-values of TWAS associations were replicable in large samples.

### Advances

Our study shows that gene-based association analyses can bridge gaps in existing neuroimaging genomic studies. Specifically, the approach begins to fill the mechanistic gap in traditional GWAS by extending these studies to identify associations of phenotypes with regionally specific gr-expression, rather than with regionally agnostic SNPs. Moreover, the approach reduces the multiple-testing burden of GWAS by orders of magnitude. Separately, the approach complements regional transcriptomic studies by extending these studies to thousands of people with available genomes.

We demonstrated the unique combination of these advantages with 3 complementary analyses. First, we showed that, like GWAS, our method separates people by genetic ancestry (**Fig 2A**). Second, we showed that, like regional transcriptomic analyses, our method separates brain regions by patterns of gr-expression (**Fig 2B**). Third, we showed strong similarities between the inter-regional correlation of gr-expression from the UK Biobank and directly assayed expression from the Allen Human Brain Atlas and the GTEx Project (**Fig 2C–2K**). In this way, we showed that our approach produces neurobiologically interpretable estimates of regionally specific gr-expression in large human populations.

Our method allowed us to directly benchmark gene-based TWAS against SNP-based GWAS. First, we showed that TWAS associations are broadly related to, but also distinct from, GWAS associations (**Fig 3C**). Second, we showed that TWAS can discover associations of many genes that are otherwise undetected with GWAS (**Fig 3D**) and that these discoveries strongly favor brain-related annotations (**Fig 3E**). Third, we showed that inter-regional associations are interpretable and further increase the utility of TWAS (**Fig 3F**). Collectively, these results directly demonstrate the conceptual and practical strengths of TWAS.

Separately, our study built on these results in 3 additional ways. First, it integrated estimates of regionally specific gr-expression with gray-matter volumes and brain-related clinical phenotypes (**Fig 4**). Second, it extended the single-gene TWAS to build polygenic models of neuroimaging phenotypes (**Fig 5**). Third, it showed that the magnitudes of TWAS associations are generally replicable but that the *p*-values of TWAS associations are highly sensitive to sample sizes (**Fig 6**). These results outline a path towards the replicable integration of polygenic gr-expression with complex neuroimaging and clinical phenotypes.

Separately, the study of gr-expression provides unique advantages over the study of directly assayed expression because it allows to focus on the stable, genetically regulated aspects of gene expression without the need to control for the potential confounders of environmental factors and acquisition biases, including batch effects [116]. Similarly, an important advantage of this study relative to group-averaged transcriptomic studies is the lack of evident bias attributable to distance effects [60]. This lack of bias arises because the associations are computed over people, rather than over brain regions. For example, while we found that the observed inter-regional correlation between estimated gr-expression and directly assayed expression could not be explained solely by distance dependence, a distance-based explanation would not invalidate our results because it would reflect biological, rather than artifactual, effects.

### Limitations

Our approach has many benefits, but it also has limitations. First, our analyses still require large samples to enable replicable associations (**Fig 6C–6E**). Nonetheless, the lower multiple-testing burden of TWAS makes this problem less acute than for tests of millions of SNPs in GWAS. Similarly, much like correlations of adjacent SNPs in GWAS, correlations of gr-expression in TWAS, while generally smaller and less common, can make it difficult to fine-map causal genes. Future studies could adopt mendelian randomization to enable causal inference, although this approach comes with its own limitations, including the difficulty of accounting for horizontal pleiotropy (the effect of one gene on unrelated phenotypes) [117–119]. Finally, our focus on genes necessarily misses the effects of variants that operate through means other than the regulation of gene expression.

Second, relative to spatially specific transcriptomic studies, our approach is restricted to a small number of brain regions. In future studies, we propose to overcome this limitation by modeling known relationships between regional and network organization [120]. We also propose that the adoption of similar models will allow researchers to integrate regional associations with high-resolution single-cell atlases of gene expression and link these associations to specific cell types [121].

Third, our results integrate genomic biobanks with available data on genetics, gene expression, neuroimaging, and clinical phenotypes. Such integration necessarily comes with the challenges of demographic diversity and matching. Our analyses were primarily based on European populations and may not necessarily generalize to other populations. As genomic, transcriptomic, neuroimaging, and clinical data continue to increase in size and scope, it will be important to extend these results to analyses of other populations.

## Conclusions

We identified associations between individual genetic variation, gene expression, neuroimaging phenotypes, and brain-related clinical phenotypes in large samples for which we cannot otherwise directly measure all these variables. Our analyses allowed us to integrate gene-level data and discover candidate mechanisms that link gr-expression via neuroimaging phenotypes to brain disorders. Collectively, these analyses demonstrate the advantages of gene-based methods in human neuroscience. Our resource can help facilitate wider adoption of these methods in future studies and thus advance the understanding of individual variation in brain organization and function.

## Methods

### Joint-Tissue Imputation models

We used Joint-Tissue Imputation models of gr-expression that were previously trained on postmortem gene expression data from GTEx. In this section, we describe the main aspects of quality control and training of these models. We refer the readers to the original studies of Joint Tissue Imputation [43] and the GTEx v8 data set [56] for a more detailed discussion of these approaches.

Joint-Tissue Imputation models estimate gr-expression as the linear combination of SNPs that are close to the gene of interest along the linear genome. The training of these models, therefore, required data on tissue-specific gene expression and whole-genome sequencing from the same people. The GTEx v8 data set included these data for brain regions of 838 donors that passed internal GTEx biospecimen quality controls [122]. The donors had the following demographics: Age, 21 to 70 years (mean 53); sex, 34% female; ancestry, 85.3% European American/12.3% African American/1.4% Asian American. The data set contained RNA-seq from ten brain regions. **Table 1** summarizes the names of these regions and the number of samples used to train models in each region. For completeness, it also summarizes our definitions of the corresponding regions in volumetric Allen Human Brain Atlas data, and in surface-based UK-Biobank and Human-Connectome Project data.

**Table 1:** GTEx regions (tissues) and corresponding definitions of volume-based and surface-based imaging parcellations.

**Name of GTEx tissue** (number of available tissue samples).	**Label names of volume-based parcellation for the Allen Human Brain Atlas gene expression.** Parcels for the substantia nigra and hypothalamus are from the subcortical atlas of Pauli abd colleagues [124]. Parcel for the cerebellar hemisphere is from the atlas of Diedrichsen and colleagues [125]. All other parcels are from the Harvard Oxford Atlas [126].	**Label names of surface-based parcellation for the UK-Biobank and Human-Connectome Project neuroimaging phenotypes.** Parcels for Brodmann Areas 9 and 24 are from the Desikan-Killiany-Tourville Atlas [127] (for the UK Biobank) or the Desikan-Killiany Atlas [128] (for the HCP). All other parcels are from the FreeSurfer subcortical segmentation [129].
DLPFC/Brodmann Area 9 (175 samples).	*Middle frontal gyrus*	*Middle frontal gyrus (rostral and caudal divisions)*
Anterior Cingulate/Brodmann Area 24 (147 samples).	*Cingulate gyrus*, *anterior division*	*Cingulate cortex (rostral anterior and caudal anterior divisions)*
Amygdala (129 samples).	*Amygdala*	*Amygdala*
Hippocampus (165 samples).	*Hippocampus*	*Hippocampus*
Caudate (194 samples).	*Caudate*	*Caudate*
Putamen (170 samples).	*Putamen*	*Putamen*
Nucleus accumbens (202 samples).	*Accumbens*	*Accumbens*
Cerebellar hemisphere (175 samples).	*Cerebellar lobules I-X*	*Cerebellum*
Substantia nigra (114 samples).	*Substantia nigra (pars compacta and pars reticulata)*	N/A
Hypothalamus (170 samples).	*Hypothalamus*	N/A

DLPFC, dorsolateral prefrontal cortex; GTEx, Genotype-Tissue Expression Project; HCP, Human Connectome Project.

The following steps were taken to maximize the predictive accuracy of estimation and to minimize confounders. First, the assayed gene expression levels were controlled for sex, sequencing platform, the top 5 principal components, as well as probabilistic estimation of expression residuals, a Bayesian model of hidden confounders [123]. Second, the models were trained only on biallelic SNPs that had a minor allele frequency of at least 0.05 and that were in Hardy–Weinberg equilibrium (*p* > 0.05), i.e., only on SNPs that had both sufficient and stable variation. Third, to reduce the effects of linkage disequilibrium, highly correlated (*r*^2^ ≥ 0.9) SNPs were pruned and the models were trained only on SNPs near the gene of interest. The optimal threshold for proximity was determined separately for each gene by cross-validation. Finally, to additionally control for overfitting, the models incorporated elastic-net regularization and the training was based on 5-fold cross-validation.

As part of the replication analysis (**S6 Fig**), we also considered Joint-Tissue Imputation models that were trained on 415 samples of sequencing and expression data from the DLPFC in the PsychENCODE project [57]. These data were processed and trained in the same way as the original study of Joint-Tissue Imputation. All pretrained models are available online at https://doi.org/10.5281/zenodo.3842289 (GTEx-trained models) and https://doi.org/10.5281/zenodo.3859065 (PsychEncode-trained models).

### Genotype-Tissue Expression Project (GTEx) and Allen Human Brain Atlas Data

Our analyses of inter-regional correlations (**Fig 2**) compared the estimated gr-expression data described in the previous section to directly assayed expression data from GTEx and the Allen Human Brain Atlas. This section describes our preprocessing of these latter data sets.

We downloaded the most recent release (v8) of the GTEx gene-expression data from https://gtexportal.org/home/downloads/adult-gtex. These data were acquired from 340 donors (an average of 199 donors per region). Gene expression levels were quantified and normalized by GTEx, and genes were selected based on expression thresholds as previously described [56].

We downloaded the Allen Human Brain Atlas microarray gene-expression data from https://human.brain-map.org/static/download. The data were acquired from 6 donors (42 ± 12 years old, 1 female). Brain-wide gene expression levels were quantified and normalized by the Allen Institute, as previously described [130].

Our preprocessing of these data followed current best practices [131]. All imputed and directly assayed expression data were normalized to have zero mean and unit variance across regions. In addition, data from the Allen Human Brain Atlas were filtered to exclude genes whose expression level did not exceed the background signal (as specified by the file PACall.csv). These data were also nonlinearly registered to reference coordinate space [132], assigned to regions with a 2 mm distance threshold, and averaged across all available probes and the left and right hemispheres.

### UK Biobank genomic and neuroimaging data

We analyzed data from 45,549 people, or all available people from the UK Biobank with genome-wide genotyping and neuroimaging volumes. Our sample had the following demographics: Age, 64 ± 7.7 years old; sex, 52% female; self-reported ethnicity, 96.7% white/0.6% black/1.1% South Asian/0.3% Chinese/0.5% Mixed/0.8% Other (S1 Table). In this section, we describe the main aspects of quality control and processing of these data by the UK Biobank. We refer the readers to the original publications [17,37,133] for a more detailed discussion of these and other questions.

Genome-wide genotype imputation was performed using data from the Haplotype Reference Consortium [134] as the main imputation reference panel, as well as merged UK10K and 1000 Genomes Phase 3 data sets as the secondary imputation reference panel [135]. The data passed an automated quality-control pipeline [37]. This pipeline comprised marker-based and sample-based quality control. Marker-based control included tests for batch effects, plate effects, deviation from Hardy–Weinberg equilibrium, sex effects, array effects, and sequencing replicability. Separately, sample-based control included tests for unusually high fractions of heterozygous or missing loci, as well as for mismatch between self-reported sex and the intensity of sex-chromosome markers.

Our TWAS (**Figs 3 and 4**) sought to minimize the confounders of genetic ancestry by focusing on the “White British” sample of the UK-Biobank cohort (39,565 people). We followed UK Biobank analyses to select people who self-reported as “White British” and who had similar genetic ancestry based on UK-Biobank principal component analysis on 147,604 genotype markers (pruned to minimized linkage disequilibrium) over 407,219 unrelated people [37]. By contrast, our analyses of genetic ancestral relationships, regional identities, and inter-regional correlations (**Fig 2**) focused on the remaining ethnically diverse sample of the UK-Biobank cohort (5,984 people).

All UK Biobank neuroimaging data were processed by the UK Biobank automated brain-imaging pipeline [133]. The pipeline flagged missing and distorted data, registered images to common reference space, and computed imaging-derived phenotypes. We used phenotypes computed on MRI scans from the first imaging visit. Our analyses included only cortical and subcortical brain regions that had available estimates of gray-matter volume and gr-expression and that passed UK Biobank quality control exclusion criteria (see the original reference [133] for a detailed discussion). Volumes of these regions were computed by the UK Biobank using FreeSurfer cortical and subcortical segmentations [61] (**Table 1**) and were averaged across the left and right hemispheres.

### Human Connectome Project genomic and neuroimaging data

The full HCP contains 1,142 people with brain-wide genotyping sequences and neuroimaging data. This cohort had the following demographics: Age 29 ± 3.7 years old, 54% females, 149 pairs of monozygotic twins. In this section, we describe our curation of these data to generate a sample of 657 people. We also describe the main aspects of quality control and processing of these data by the HCP, and our estimation of phenotypes from these data. We refer the readers to the original HCP publications [136–139] for an additional extensive discussion of quality control and data processing.

Genotyping of all people was derived from blood or saliva samples. The genotype data comprised probabilities of single-nucleotide variants, estimated using the Illumina Multi-Ethnic Global Array. Quality-control procedures of these data included verification of self-reported common ancestry for siblings, as well as zygosity for twins.

As in our analyses of the UK Biobank, we sought to minimize the confounders of genetic ancestry by focusing our association analyses on a sample of 657 non-twins of European genetic ancestry. We estimated genetic ancestry using the principal components of genotyping data from this cohort, computed with EIGENSTRAT [140]. We defined people to be of European ancestry when they self-reported as European and when they had similar genetic ancestry based on principal-component structure [37]. Finally, we randomly removed a single person from all pairs of monozygotic twins.

We analyzed structural and resting-state functional MRI phenotypes from the HCP. All data were processed using the HCP minimal preprocessing pipeline [137] and were passed through a standardized quality control pipeline [138]. Structural MRI acquisitions were initially reviewed for image blurriness, motion, and other artifacts. Volumes of these regions were then estimated using FreeSurfer cortical and subcortical segmentations [61]. FreeSurfer-based reconstructions were inspected for obvious errors. Separately, resting-state functional MRI acquisitions were scored for 9 quality control measures that centered on the temporal signal-to-noise ratio, image smoothness, as well as the extent of absolute and relative head motion. The data were registered with MSM-All [141] and denoised with ICA-FIX [139].

In our study, we computed 3 functional MRI phenotypes on these data. First, we computed the amplitude of low-frequency fluctuations as the total power of spontaneous intra-regional activity within the 0.01 to 0.08 Hz range. Second, we computed regional homogeneity as the mean Pearson correlation between all pairs of intra-regional voxels. Third, we computed mean coactivity as the mean Pearson correlation between the activity of the region and all other regions of interest.

Individual variation in homogeneity and coactivity strongly correlated with individual variation of the global signal, the mean activity of all brain voxels. This variation can reflect artifact but also aspects of vigilance and non-neuronal physiology [142]. To focus on correlation structure unaffected by such properties, we computed these phenotypes after regressing out the global signal from voxel time series (for homogeneity) or regional time series (for coactivity).

We computed each phenotype separately for each scan of each person and then averaged the phenotypes across the 4 available scans and the left and right hemispheres.

### Analyses of genetic ancestry, regional identity, and inter-regional correlation structure

We created principal component embeddings of ancestral and regional gr-expression for the ethnically diverse sample of the UK-Biobank cohort. We first constructed a 3D array of 5,984 people × 1,892 genes × 10 regions, where people comprised the UK-Biobank sample (**S1 Table**), and genes comprised all genes with available estimates of gr-expression in all the 10 GTEx regions. We then analyzed reshaped versions of this array. First, to extract ancestral structure, we computed principal components of the 5,984 × 18,920 matrix of brain-wide gr-expression across people. Second, to extract regional structure, we computed principal components of the 59,840 × 1,892 matrix of region-specific gr-expression across people.

We likewise compared inter-regional correlations of expression on subsets of genes that simultaneously had available expression in the UK-Biobank, GTEx, and Allen Human Brain Atlas data. These subsets ranged from 2,837 to 4,220 genes (median 3,642) and differed for each region of interest because each region had a distinct set of available gr-expression models. For all pairs of regions, we computed inter-regional coexpression using the subsets of genes that had expression data in both regions. Finally, we averaged the inter-regional coexpression matrices across all people in each data set.

To test for distance effects, we computed Spearman correlation coefficients between inter-regional coexpression and Euclidean distance between centroids of regions in the volume-based parcellation (**Table 1**). To test the effects of distance on the similarity of inter-regional coexpression, we generated 10,000 coexpression matrices with permuted ranks and empirical Spearman correlations with Euclidean distance.

### Association of genetically regulated gene expression with neuroimaging phenotypes

#### Transcriptome-wide association studies (TWAS)

We estimated associations of gr-expression and neuroimaging phenotypes using ordinary least square regression models, with covariates of genetic ancestry, sex, and age. We followed common practices for addressing population stratification by modeling genetic ancestry by the top 40 principal components of the genotypes in each sample. We used the precomputed principal components for the UK Biobank [37] and EIGENSTRAT [140] to compute principal components for the HCP. We tested associations between the gr-expression and volume of the same region (intra-regional TWAS, **S2 Table**) and between the gr-expression of one region and the volumes of other regions (inter-regional TWAS, **S4 Table**).

#### Genome-wide association studies (GWAS)

We used REGENIE [143] to perform GWAS on the regional gray-matter volumes for the white-British sample of the UK-Biobank cohort. REGENIE is a machine-learning method for fitting genome-wide regressions to complex phenotypes, particularly for large samples with multiple phenotypes of interest. We first filtered (directly genotyped and imputed) autosomal SNPs with a minor allele frequency greater than 1% and an information score greater than 0.2 based on the full UK-Biobank cohort of roughly 500,000 people (information score denotes the fraction of data at an imputed marker that approximately equates to perfectly observed genotype calls [37]). We then set imputed SNPs using a hard-call threshold of 0.01 and, with respect to our sample of interest, filtered the SNPs with a minor allele frequency greater than 1%, missingness less than 5%, and Hardy–Weinberg equilibrium test *p* < 10^−5^. We performed GWAS on the remaining 8,072,589 SNPs, after regressing out age, sex, and the top 40 principal components.

#### Comparison of genome-wide and transcriptome-wide associations

Joint-Tissue Imputation models of gene gr-expression can, in general, contain several SNPs. Similarly, one SNP can, in general, be part of several gr-expression models. We used this knowledge to map GWAS-derived SNP associations to TWAS-derived gene associations (**Fig 3C**). We made this mapping using 2 approaches:

*Many-to-many mapping*: In this mapping, we linked each TWAS-derived gene association with all SNPs that comprised the gr-expression model of that gene. Equivalently, we linked each GWAS-derived SNP association to all the gr-expression models of which that SNP was a part.

*One-to-one mapping*: We next filtered the many-to-many mapping in 2 steps. First, we filtered TWAS-derived gene associations to preserve links to the strongest available GWAS-derived SNP association. Second, we filtered the remaining GWAS-derived SNP associations to preserve links to the strongest remaining TWAS-derived gene association. This two-step filtering of many-to-many associations therefore guaranteed one-to-one relationships.

#### Polygenic modeling association studies

We modeled associations between polygenic gr-expression and phenotypes with covariates of genetic ancestry, sex, and age. First, we selected genes that had nominal TWAS associations with phenotypes (*p* < 0.001, uncorrected). Second, we averaged the normalized gr-expression of these selected genes (reversing the sign of gr-expression that had negative associations). Third, we computed the Pearson correlation coefficient between these averaged gr-expression and phenotypes. Finally, we repeated this process 10,000 times on data that had randomized (permuted) assignment of phenotypes to subjects, but the same values of individual phenotypes and gr-expression.

### Gene-set enrichment analyses

We performed gene-set enrichment analysis for biological annotations from the NHGRI-EBI GWAS catalog [75] (**Fig 3E**). Each phenotype in this catalog is situated within the Experimental Factor Ontology, a general ontology that includes terms from multiple more specialized ontologies and describes a wide range of measurements, including healthy and diseased phenotypes. All annotations reflect findings from curated GWAS analyses.

We used a semi-automated pipeline to detect brain-related terms in this ontology in 2 steps. In the first step, we flagged each term as brain-related if words in its ontology tree included at least one of the following word segments: nerv, neur, cogn, psyc, ment, brai. Second, 2 authors (NH and MR) manually and independently checked these candidate terms to confirm or exclude their brain-related nature. **S3 Table** lists the phenotypes that were enriched for TWAS genes at *p*_FDR_ < 0.05 and also lists their brain-relatedness indicator.

We performed gene-set enrichment analyses for clinical phenotypes in the BioVU TWAS, a database of associations between genetically regulated gene expression and clinical phenotypes derived from the Vanderbilt Biobank. For these analyses, we considered 70,439 people of European ancestry. Phenotypes were represented as phenotype codes based on ICD-9 codes [144]. We restricted our analyses to *mental disorders* and *neurological* phenotype code categories and included all gene-phenotype pairs that showed associations in the BioVU TWAS at *p*_FDR_ < 0.05.

We performed gene-set enrichment analyses using WebGestalt [145,146]. In both cases, we used a hypergeometric null model to test the enrichment of genes that had TWAS associations of *p*_FDR_ < 0.05 against a reference set of all genes in the TWAS (in other words, against all genes with relevant gr-expression models that passed baseline performance thresholds).

### Interactive application

We developed a browser-based application for interactive analysis of our association results. This application is available on GitHub at https://github.com/nhunghoang/twas-webapp.

## Supporting information

S1 Fig UK Biobank TWAS results for all considered brain regions. Left. TWAS of gr-expression and brain volumes for all regions. Each point denotes an association between the individual variation of gr-expression of a gene and volume in the same region. The horizontal axis denotes the chromosome location of individual genes. The vertical axis denotes–log_10_ *p*-values. Solid-color points represent associations that passed *p*_FDR_ = 0.05 or *p*_Bonferroni_ = 0.05 (horizontal lines). **Right.** Associations between SNP-based GWAS and gene-based TWAS for all regions. Left: Scatter plots of *p-*values (–log_10_ *p*) for associations of all genes and SNPs. These plots preserve all genes and SNPs but lack the one-to-one relationship between genes and SNPs. Right: Corresponding scatter plots of the best-performing genes and SNPs. Each gene in TWAS matches with its best-performing SNP in GWAS. Similarly, each SNP in GWAS matches with its best-performing gene in TWAS. These plots show one-to-one relationships but exclude many genes and SNPs.
(TIFF)

S2 Fig Effects of Bonferroni correction on enrichment analyses and inter-regional TWAS. **(A, B)** Effects of Bonferroni correction on enrichment analyses in the NHGRI-EBI Catalog. **(A)** Enrichment for biological annotations of genes whose gr-expression predicted regional volumes (*p*_Bonferroni_ < 0.05). Each point represents a biological annotation associated with at least 1 gene. The horizontal axis denotes the *p*-values (–log_10_ *p*_FDR_) of individual annotations. **(B)** Comparison of *p*_FDR_ for biological annotations of genes whose gr-expression predicted regional volumes under FDR and Bonferroni corrections. **(C, D)** Effects of Bonferroni correction on inter-regional associations between gr-expression and regional brain volumes. **(C)** Absolute numbers of associations. Numbers of genes whose gr-expression in one region (columns) predicted (*p*_Bonferroni_ < 0.05) the volume of another region (rows). **(D)** Overlap coefficients. Number of genes that were common to both intra-regional and inter-regional associations in **C**, normalized by the size of the smaller of the intra- and inter-regional gene sets.
(TIFF)

S3 Fig Effects of Bonferroni correction on associations of gr-expression with both neuroimaging and clinical phenotypes. **(A)** Heatmap showing the number of times by which genes (rows) with regional gr-expression (columns) were linked to both regional volumes and clinical phenotypes. Each count denotes a regional gr-expression that was associated with both a regional volume in the UK Biobank TWAS and with a brain-related clinical phenotype in the BioVU TWAS (*p*_Bonferroni_ < 0.05). **(B)** Heatmap showing the number of genes with regional gr-expression that linked regional volumes (columns) with clinical phenotypes (rows). Each count denotes a regional gr-expression that was associated with both a regional volume in the UK Biobank TWAS and with a brain-related clinical phenotype in the BioVU TWAS (*p*_Bonferroni_ < 0.05). **(C)** Enrichment of clinical phenotypes for genes whose gr-expression predicted regional volumes (rows) in the UK Biobank TWAS (*p*_Bonferroni_ < 0.05). Each point represents a brain-related clinical phenotype associated with at least 1 gene. The horizontal axis denotes the *p*-values (–log_10_ *p*_FDR_) of individual phenotypes. **(D)** Comparison of *p*_FDR_ for clinical phenotypes of genes whose gr-expression predicted regional volumes under FDR and Bonferroni corrections.
(TIFF)

S4 Fig Associations of polygenic gr-expression with neuroimaging phenotypes. Scatter plots of polygenic gr-expression and neuroimaging phenotypes. The horizontal axis shows values of observed phenotypes, and the vertical axis denotes values of polygenic gr-expression. Points represent single individuals.
(TIFF)

S5 Fig Association of gene numbers with *r*-values and *p*-values in polygenic models. Scatter plots showing the number of genes in each polygenic model and model *r*-values and *p-*values (–log_10_ *p*_FDR_ from permutation testing). Each plot shows a distinct phenotype. Colors denote brain regions as in **Fig 5**.
(TIFF)

S6 Fig Replicability of estimated genetically regulated gene expression and TWAS. **(A) Left.** Within-regional associations of gr-expression and gray-matter volumes for the DLPFC, based on gr-expression models trained on GTEx and PsychEncode data. Each point denotes an association between the individual variation of gr-expression and volume in the same region. The horizontal axis shows the chromosome location of individual genes. The vertical axis shows the *p-*values (–log_10_ *p*) of associations. Solid-color points show associations that passed *p*_FDR_ = 0.05 or *p*_Bonferroni_ = 0.05 (horizontal lines). **(A) Right.** Associations between SNP-based GWAS and gene-based TWAS for the DLPFC, based on gr-expression models trained on GTEx and PsychEncode data. Left: Scatter plots of *p-*values (–log_10_ *p*) for associations of all genes and SNPs. These plots preserve all genes and SNPs but lack the one-to-one relationship between genes and SNPs. Right: Corresponding scatter plots for the best-performing genes and SNPs. Each gene in TWAS matches with its best-performing SNP in GWAS. Similarly, each SNP in GWAS matches with its best-performing gene in TWAS. These plots show one-to-one relationships but exclude many genes and SNPs. **(B)** Scatter plots of effect magnitudes and *p*-values (–log_10_ *p*) for the UK Biobank TWAS of regional gray-matter volumes. Dots denote associations for all genes from the TWAS. Note the double-logarithmic scale. **(C)** Correlations between effect magnitudes of all gene associations in the replication and discovery TWAS of regional gray-matter volumes. Dots denote analyses on the full UK Biobank (discovery) and HCP (replication) samples. Box plots denote analyses of discovery-replication splits of the white-British UK-Biobank sample, ordered from small to large replication samples. Each box plot was estimated from 300 random splits of the white-British UK-Biobank sample. **Fig 6D** shows a similar plot, but filtered to include only genes that passed *p*_FDR_ < 0.05 in the discovery TWAS.
(TIFF)

S1 Table Demographics of the UK Biobank samples. Demographics of the diverse-ancestry and White-British samples of the UK Biobank.
(XLSX)

S2 Table Summary of intra-regional associations in the UK Biobank TWAS. All associations from **Figs 3B** and **S1** that passed *p*_FDR_ = 0.05, ordered by region name, then *p*_FDR_.
(XLSX)

S3 Table Summary of enrichment analyses for biological annotations in the NHGRI-EBI Catalog. All enrichment associations from **Fig 3E** that passed *p*_FDR_ = 0.05, grouped by brain-relatedness, annotation, and region volume.
(XLSX)

S4 Table Summary of inter-regional associations in the UK Biobank TWAS. All interregional associations from **Fig 3G** that passed *p*_FDR_ = 0.05, ordered by region name, then *p*_FDR_.
(XLSX)

S5 Table Summary of enrichment analyses for brain-related clinical phenotypes in the BioVU Catalog. All enrichment associations from **Fig 4D** that passed *p*_FDR_ = 0.05, ordered by *p*_FDR_ of TWAS.
(XLSX)

S1 Data Association of polygenic gr-expression with neuroimaging phenotypes (Fig 5C). This data set (hdf5 file) contains arrays for reproducing associations in **Fig 5C**. It specifically contains single-gene correlations (file key twas-pearsons), poly-gene correlations (file key poly-pearsons), permutation-test correlations (file key poly-null-pearsons), and order of regional phenotype in these arrays (file key reg-phen-order).
(HDF5)

S2 Data Replication of effects and *p*-values (Fig 6C and 6D). This data table contains the replication fractions of discovery TWAS genes in **Fig 6C** (column replication_fraction). It also contains the correlations between effect magnitudes for these discovery-replication TWAS pairings in **Fig 6D** (column effect_spearman). TWAS are identifiable by their regional volume phenotype of interest (column region), the replication cohort and size (column cohort), and the random-sample iteration (column iteration).
(CSV)

## Abbreviations

DLPFC: dorsolateral prefrontal cortex
FDR: false discovery rate
GTEx: Genotype-Tissue Expression Project
GWAS: genome-wide association studies
HCP: Human Connectome Project
SNP: single-nucleotide polymorphism
TWAS: transcriptome-wide association studies

## References

[1] Dubois J, Adolphs R. Building a Science of Individual Differences from fMRI. Trends Cogn Sci. 2016;20:425–443. doi: 10.1016/j.tics.2016.03.014 27138646 PMC4886721

[2] Zilles K, Amunts K. Individual variability is not noise. Trends Cogn Sci. 2013;17:153–155. doi: 10.1016/j.tics.2013.02.003 23507449

[3] Genon S, Eickhoff SB, Kharabian S. Linking interindividual variability in brain structure to behaviour. Nat Rev Neurosci. 2022;23:307–318. doi: 10.1038/s41583-022-00584-7 35365814

[4] Sun L, Liang X, Duan D, Liu J, Chen Y, Wang X, et al. Structural insight into the individual variability architecture of the functional brain connectome. Neuroimage. 2022;259:119387. doi: 10.1016/j.neuroimage.2022.119387 35752416

[5] Gordon EM. Individual Variability of the System-Level Organization of the Human Brain. Cereb Cortex. 2015;bhv239. doi: 10.1093/cercor/bhv239 26464473 PMC5939195

[6] Mueller S, Wang D, Fox MD, Yeo BTT, Sepulcre J, Sabuncu MR, et al. Individual Variability in Functional Connectivity Architecture of the Human Brain. Neuron. 2013;77:586–595. doi: 10.1016/j.neuron.2012.12.028 23395382 PMC3746075

[7] Mills KL, Siegmund KD, Tamnes CK, Ferschmann L, Wierenga LM, Bos MGN, et al. Inter-individual variability in structural brain development from late childhood to young adulthood. Neuroimage. 2021;242. doi: 10.1016/j.neuroimage.2021.118450 34358656 PMC8489572

[8] Cui Z, Li H, Xia CH, Larsen B, Adebimpe A, Baum GL, et al. Individual Variation in Functional Topography of Association Networks in Youth. Neuron. 2020;106. doi: 10.1016/j.neuron.2020.01.029 32078800 PMC7182484

[9] Finn ES, Todd Constable R. Individual variation in functional brain connectivity: implications for personalized approaches to psychiatric disease. Dialogues Clin Neurosci. 2016;18:277–287. doi: 10.31887/DCNS.2016.18.3/efinn 27757062 PMC5067145

[10] Smith SM, Nichols TE, Vidaurre D, Winkler AM, Behrens TEJ, Glasser MF, et al. A positive-negative mode of population covariation links brain connectivity, demographics and behavior. Nat Neurosci. 2015;18:1565–1567. doi: 10.1038/nn.4125 26414616 PMC4625579

[11] Finn ES, Shen X, Scheinost D, Rosenberg MD, Huang J, Chun MM, et al. Functional connectome fingerprinting: Identifying individuals using patterns of brain connectivity. Nat Neurosci. 2015;18. doi: 10.1038/nn.4135 26457551 PMC5008686

[12] Gordon EM, Laumann TO, Gilmore AW, Newbold DJ, Greene DJ, Berg JJ, et al. Precision Functional Mapping of Individual Human Brains. Neuron. 2017;95. doi: 10.1016/j.neuron.2017.07.011 28757305 PMC5576360

[13] Fan Y-S, Li L, Peng Y, Li H, Guo J, Li M, et al. Individual-specific functional connectome biomarkers predict schizophrenia positive symptoms during adolescent brain maturation. Hum Brain Mapp. 2021;42:1475–1484. doi: 10.1002/hbm.25307 33289223 PMC7927287

[14] Michon KJ, Khammash D, Simmonite M, Hamlin AM, Polk TA. Person-specific and precision neuroimaging: Current methods and future directions. Neuroimage. 2022;263:119589. doi: 10.1016/j.neuroimage.2022.119589 36030062

[15] Hawrylycz M, Miller JA, Menon V, Feng D, Dolbeare T, Guillozet-Bongaarts AL, et al. Canonical genetic signatures of the adult human brain. Nat Neurosci. 2015;18. doi: 10.1038/nn.4171 26571460 PMC4700510

[16] Hibar DP, Stein JL, Renteria ME, Arias-Vasquez A, Desrivières S, Jahanshad N, et al. Common genetic variants influence human subcortical brain structures. Nature. 2015;520. doi: 10.1038/nature14101 25607358 PMC4393366

[17] Elliott LT, Sharp K, Alfaro-Almagro F, Shi S, Miller KL, Douaud G, et al. Genome-wide association studies of brain imaging phenotypes in UK Biobank. Nature. 2018;562. doi: 10.1038/s41586-018-0571-7 30305740 PMC6786974

[18] Zhao B, Luo T, Li T, Li Y, Zhang J, Shan Y, et al. Genome-wide association analysis of 19,629 individuals identifies variants influencing regional brain volumes and refines their genetic co-architecture with cognitive and mental health traits. Nat Genet. 2019;51. doi: 10.1038/s41588-019-0516-6 31676860 PMC6858580

[19] Whitaker KJ, Vertes PE, Romero-Garcia R, Vasa F, Moutoussis M, Prabhu G, et al. Adolescence is associated with genomically patterned consolidation of the hubs of the human brain connectome. Proc Natl Acad Sci U S A. 2016;113:9105–9110. doi: 10.1073/pnas.1601745113 27457931 PMC4987797

[20] Krienen FM, Yeo BTT, Ge T, Buckner RL, Sherwood CC. Transcriptional profiles of supragranular-enriched genes associate with corticocortical network architecture in the human brain. Proc Natl Acad Sci U S A. 2016;113:E469–E478. doi: 10.1073/pnas.1510903113 26739559 PMC4739529

[21] Burt JB, Demirtaş M, Eckner WJ, Navejar NM, Ji JL, Martin WJ, et al. Hierarchy of transcriptomic specialization across human cortex captured by structural neuroimaging topography. Nat Neurosci. 2018;21:1251–1259. doi: 10.1038/s41593-018-0195-0 30082915 PMC6119093

[22] Reardon PK, Seidlitz J, Vandekar S, Liu S, Patel R, Park MTM, et al. Normative brain size variation and brain shape diversity in humans. Science (1979). 2018;360:1222–1227. doi: 10.1126/science.aar2578 29853553 PMC7485526

[23] Anderson KM, Krienen FM, Choi EY, Reinen JM, Yeo BTT, Holmes AJ. Gene expression links functional networks across cortex and striatum. Nat Commun. 2018;9:1428. doi: 10.1038/s41467-018-03811-x 29651138 PMC5897339

[24] Vogel JW, La Joie R, Grothe MJ, Diaz-Papkovich A, Doyle A, Vachon-Presseau E, et al. A molecular gradient along the longitudinal axis of the human hippocampus informs large-scale behavioral systems. Nat Commun. 2020;11:960. doi: 10.1038/s41467-020-14518-3 32075960 PMC7031290

[25] Richiardi J, Altmann A, Milazzo A-C, Chang C, Chakravarty MM, Banaschewski T, et al. Correlated gene expression supports synchronous activity in brain networks. Science. 2015;1979(348):1241–1244. doi: 10.1126/science.1255905 26068849 PMC4829082

[26] Wang G-Z, Belgard TG, Mao D, Chen L, Berto S, Preuss TM, et al. Correspondence between Resting-State Activity and Brain Gene Expression. Neuron. 2015;88:659–666. doi: 10.1016/j.neuron.2015.10.022 26590343 PMC4694561

[27] Berto S, Treacher AH, Caglayan E, Luo D, Haney JR, Gandal MJ, et al. Association between resting-state functional brain connectivity and gene expression is altered in autism spectrum disorder. Nat Commun. 2022;13:3328. doi: 10.1038/s41467-022-31053-5 35680911 PMC9184501

[28] Zhao B, Li T, Smith SM, Xiong D, Wang X, Yang Y, et al. Common variants contribute to intrinsic human brain functional networks. Nat Genet. 2022;54. doi: 10.1038/s41588-022-01039-6 35393594 PMC11987081

[29] Stein JL, Medland SE, Vasquez AA, Hibar DP, Senstad RE, Winkler AM, et al. Identification of common variants associated with human hippocampal and intracranial volumes. Nat Genet. 2012;44. doi: 10.1038/ng.2250 22504417 PMC3635491

[30] van der Meer D, Frei O, Kaufmann T, Shadrin AA, Devor A, Smeland OB, et al. Understanding the genetic determinants of the brain with MOSTest. Nat Commun. 2020;11. doi: 10.1038/s41467-020-17368-1 32665545 PMC7360598

[31] Smith SM, Douaud G, Chen W, Hanayik T, Alfaro-Almagro F, Sharp K, et al. An expanded set of genome-wide association studies of brain imaging phenotypes in UK Biobank. Nat Neurosci. 2021;24. doi: 10.1038/s41593-021-00826-4 33875891 PMC7610742

[32] Grasby KL, Jahanshad N, Painter JN, Colodro-Conde L, Bralten J, Hibar DP, et al. The genetic architecture of the human cerebral cortex. Science. 2020;1979:367. doi: 10.1126/science.aay6690 32193296 PMC7295264

[33] Thompson PM, Stein JL, Medland SE, Hibar DP, Vasquez AA, Renteria ME, et al. The ENIGMA Consortium: Large-scale collaborative analyses of neuroimaging and genetic data. Brain Imaging Behav. 2014;8. doi: 10.1007/s11682-013-9269-5 24399358 PMC4008818

[34] Thompson PM, Jahanshad N, Ching CRK, Salminen LE, Thomopoulos SI, Bright J, et al. ENIGMA and global neuroscience: A decade of large-scale studies of the brain in health and disease across more than 40 countries. Transl Psychiatry. 2020;10:100. doi: 10.1038/s41398-020-0705-1 32198361 PMC7083923

[35] Sudlow C, Gallacher J, Allen N, Beral V, Burton P, Danesh J, et al. UK Biobank: An Open Access Resource for Identifying the Causes of a Wide Range of Complex Diseases of Middle and Old Age. PLoS Med. 2015;12. doi: 10.1371/journal.pmed.1001779 25826379 PMC4380465

[36] Miller KL, Alfaro-Almagro F, Bangerter NK, Thomas DL, Yacoub E, Xu J, et al. Multimodal population brain imaging in the UK Biobank prospective epidemiological study. Nat Neurosci. 2016;19. doi: 10.1038/nn.4393 27643430 PMC5086094

[37] Bycroft C, Freeman C, Petkova D, Band G, Elliott LT, Sharp K, et al. The UK Biobank resource with deep phenotyping and genomic data. Nature. 2018;562:203–209. doi: 10.1038/s41586-018-0579-z 30305743 PMC6786975

[38] Casey BJ, Cannonier T, Conley MI, Cohen AO, Barch DM, Heitzeg MM, et al. The Adolescent Brain Cognitive Development (ABCD) study: Imaging acquisition across 21 sites. Dev Cogn Neurosci. 2018;32:43–54. doi: 10.1016/j.dcn.2018.03.001 29567376 PMC5999559

[39] Lee JJ, Wedow R, Okbay A, Kong E, Maghzian O, Zacher M, et al. Gene discovery and polygenic prediction from a genome-wide association study of educational attainment in 1.1 million individuals. Nat Genet. 2018;50:1112–1121. doi: 10.1038/s41588-018-0147-3 30038396 PMC6393768

[40] Evangelou E, Warren HR, Mosen-Ansorena D, Mifsud B, Pazoki R, Gao H, et al. Genetic analysis of over 1 million people identifies 535 new loci associated with blood pressure traits. Nat Genet. 2018;50:1412–1425. doi: 10.1038/s41588-018-0205-x 30224653 PMC6284793

[41] Yengo L, Vedantam S, Marouli E, Sidorenko J, Bartell E, Sakaue S, et al. A saturated map of common genetic variants associated with human height. Nature. 2022;610:704–712. doi: 10.1038/s41586-022-05275-y 36224396 PMC9605867

[42] Marek S, Tervo-Clemmens B, Calabro FJ, Montez DF, Kay BP, Hatoum AS, et al. Reproducible brain-wide association studies require thousands of individuals. Nature. 2022;603:654–660. doi: 10.1038/s41586-022-04492-9 35296861 PMC8991999

[43] Zhou D, Jiang Y, Zhong X, Cox NJ, Liu C, Gamazon ER. A unified framework for joint-tissue transcriptome-wide association and Mendelian randomization analysis. Nat Genet. 2020;52. doi: 10.1038/s41588-020-0706-2 33020666 PMC7606598

[44] Mai J, Lu M, Gao Q, Zeng J, Xiao J. Transcriptome-wide association studies: recent advances in methods, applications, and available databases. Commun Biol. 2023;6:899. doi: 10.1038/s42003-023-05279-y 37658226 PMC10474133

[45] Li B, Ritchie MD. From GWAS to Gene: Transcriptome-Wide Association Studies and Other Methods to Functionally Understand GWAS Discoveries. Preprint. 2021. doi: 10.3389/fgene.2021.713230 34659337 PMC8515949

[46] Wainberg M, Sinnott-Armstrong N, Mancuso N, Barbeira AN, Knowles DA, Golan D, et al. Opportunities and challenges for transcriptome-wide association studies. Nat Genet. 2019;51:592–599. doi: 10.1038/s41588-019-0385-z 30926968 PMC6777347

[47] Lu M, Zhang Y, Yang F, Mai J, Gao Q, Xu X, et al. TWAS Atlas: a curated knowledgebase of transcriptome-wide association studies. Nucleic Acids Res. 2023;51:D1179–D1187. doi: 10.1093/nar/gkac821 36243959 PMC9825460

[48] Bhattacharya A, Hirbo JB, Zhou D, Zhou W, Zheng J, Kanai M, et al. Best practices for multi-ancestry, meta-analytic transcriptome-wide association studies: Lessons from the Global Biobank Meta-analysis Initiative. Cell Genomics. 2022;2:100180. doi: 10.1016/j.xgen.2022.100180 36341024 PMC9631681

[49] Zhao B, Shan Y, Yang Y, Yu Z, Li T, Wang X, et al. Transcriptome-wide association analysis of brain structures yields insights into pleiotropy with complex neuropsychiatric traits. Nat Commun. 2021;12:2878. doi: 10.1038/s41467-021-23130-y 34001886 PMC8128893

[50] Yao S, Zhang X, Zou S-C, Zhu Y, Li B, Kuang W-P, et al. A transcriptome-wide association study identifies susceptibility genes for Parkinson’s disease. NPJ Parkinsons Dis. 2021;7:79. doi: 10.1038/s41531-021-00221-7 34504106 PMC8429416

[51] Seidlitz J, Mallard TT, Vogel JW, Lee YH, Warrier V, Ball G, et al. The molecular genetic landscape of human brain size variation. Cell Rep. 2023;42:113439. doi: 10.1016/j.celrep.2023.113439 37963017 PMC11694216

[52] Bledsoe X, Gamazon ER. A transcriptomic atlas of the human brain reveals genetically determined aspects of neuropsychiatric health. Am J Hum Genet. 2024. doi: 10.1016/j.ajhg.2024.06.002 38925120 PMC11339608

[53] Roden DM, Pulley JM, Basford MA, Bernard GR, Clayton EW, Balser JR, et al. Development of a large-scale de-identified DNA biobank to enable personalized medicine. Clin Pharmacol Ther. 2008;84. doi: 10.1038/clpt.2008.89 18500243 PMC3763939

[54] Van Essen DC, Smith SM, Barch DM, Behrens TEJ, Yacoub E, Ugurbil K. The WU-Minn Human Connectome Project: An overview. Neuroimage. 2013;80. doi: 10.1016/j.neuroimage.2013.05.041 23684880 PMC3724347

[55] Gamazon ER, Wheeler HE, Shah KP, Mozaffari SV, Aquino-Michaels K, Carroll RJ, et al. A gene-based association method for mapping traits using reference transcriptome data. Nat Genet. 2015;47:1091–1098. doi: 10.1038/ng.3367 26258848 PMC4552594

[56] Aguet F, Barbeira AN, Bonazzola R, Brown A, Castel SE, Jo B, et al. The GTEx Consortium atlas of genetic regulatory effects across human tissues. Science. 2020;1979:369. doi: 10.1126/SCIENCE.AAZ1776 32913098 PMC7737656

[57] Akbarian S, Liu C, Knowles JA, Vaccarino FM, Farnham PJ, Crawford GE, et al. The PsychENCODE project. Nat Neurosci. 2015;18:1707–1712. doi: 10.1038/nn.4156 26605881 PMC4675669

[58] Duforet-Frebourg N, Luu K, Laval G, Bazin E, Blum MGB. Detecting genomic signatures of natural selection with principal component analysis: Application to the 1000 genomes data. Mol Biol Evol. 2016;33. doi: 10.1093/molbev/msv334 26715629 PMC4776707

[59] Fulcher BD, Fornito A. A transcriptional signature of hub connectivity in the mouse connectome. Proc Natl Acad Sci U S A. 2016;113:1435–1440. doi: 10.1073/pnas.1513302113 26772314 PMC4747775

[60] Fulcher BD, Arnatkeviciute A, Fornito A. Overcoming false-positive gene-category enrichment in the analysis of spatially resolved transcriptomic brain atlas data. Nat Commun. 2021;12:1–13. 33976144 10.1038/s41467-021-22862-1 PMC8113439

[61] Fischl B. FreeSurfer. Neuroimage. 2012;62:774–781. doi: 10.1016/j.neuroimage.2012.01.021 22248573 PMC3685476

[62] Yu F, Guan Z, Zhuo M, Sun L, Zou W, Zheng Z, et al. Further identification of NSF* as an epilepsy related gene. Mol Brain Res. 2002;99. doi: 10.1016/S0169-328X(01)00345-X 11978405

[63] Cheng W, van der Meer D, Parker N, Hindley G, O’Connell KS, Wang Y, et al. Shared genetic architecture between schizophrenia and subcortical brain volumes implicates early neurodevelopmental processes and brain development in childhood. Mol Psychiatry. 2022;27. doi: 10.1038/s41380-022-01751-z 36100668

[64] Ma C, Li Y, Li X, Liu J, Luo XJ. Identification of a functional SNP rs7304782 at schizophrenia risk locus 12q24.31 and validation of its association with schiz ophrenia in Chinese populations. Psychiatry Res. 2020;294. doi: 10.1016/j.psychres.2020.113491 33070109

[65] Zhang C, Li X, Zhao L, Guo W, Deng W, Wang Q, et al. Brain transcriptome-wide association study implicates novel risk genes underlying schizophrenia risk. Psychol Med. 2023. doi: 10.1017/S0033291723000417 37092861

[66] Gouveia C, Gibbons E, Dehghani N, Eapen J, Guerreiro R, Bras J. Genome-wide association of polygenic risk extremes for Alzheimer’s disease in the UK Biobank. Sci Rep. 2022;12. doi: 10.1038/s41598-022-12391-2 35589863 PMC9120074

[67] Li QS, De Muynck L. Differentially expressed genes in Alzheimer’s disease highlighting the roles of microglia genes including OLR1 and astrocyte gene CDK2AP1. Brain Behav Immun Health. 2021;13. doi: 10.1016/j.bbih.2021.100227 34589742 PMC8474442

[68] Fernandez MV, Budde JP, Eteleeb A, Wang F, Martinez R, Norton J, et al. Functional exploration of AGFG2, a novel player in the pathology of Alzheimer disease. Alzheimers Dement. 2021;17. doi: 10.1002/alz.054240

[69] Ripke S, Neale BM, Corvin A, Walters JTR, Farh KH, Holmans PA, et al. Biological insights from 108 schizophrenia-associated genetic loci. Nature. 2014;511. doi: 10.1038/nature13595 25056061 PMC4112379

[70] Kalkman HO. Potential opposite roles of the extracellular signal-regulated kinase (ERK) pathway in autism spectrum and bipolar disorders. Preprint. 2012. doi: 10.1016/j.neubiorev.2012.07.008 22884480

[71] Iossifov I, Ronemus M, Levy D, Wang Z, Hakker I, Rosenbaum J, et al. De Novo Gene Disruptions in Children on the Autistic Spectrum. Neuron. 2012;74. doi: 10.1016/j.neuron.2012.04.009 22542183 PMC3619976

[72] Li X, Su X, Liu J, Li H, Li M, Li W, et al. Transcriptome-wide association study identifies new susceptibility genes and pathways for depression. Transl Psychiatry. 2021;11. doi: 10.1038/s41398-021-01411-w 34021117 PMC8140098

[73] Ripke S, Sanders AR, Kendler KS, Levinson DF, Sklar P, Holmans PA, et al. Genome-wide association study identifies five new schizophrenia loci. Nat Genet. 2011;43. doi: 10.1038/ng.940 21926974 PMC3303194

[74] Smoller JW, Kendler KK, Craddock N, Lee PH, Neale BM, Nurnberger JN, et al. Identification of risk loci with shared effects on five major psychiatric disorders: A genome-wide analysis. Lancet. 2013;381. doi: 10.1016/S0140-6736(12)62129-1 23453885 PMC3714010

[75] Buniello A, MacArthur JAL, Cerezo M, Harris LW, Hayhurst J, Malangone C, et al. The NHGRI-EBI GWAS Catalog of published genome-wide association studies, targeted arrays and summary statistics 2019. Nucleic Acids Res. 2019;47:D1005–D1012. doi: 10.1093/nar/gky1120 30445434 PMC6323933

[76] Donlon TA, Morris BJ, Chen R, Masaki KH, Allsopp RC, Willcox DC, et al. FOXO3 longevity interactome on chromosome 6. Aging Cell. 2017;16. doi: 10.1111/acel.12625 28722347 PMC5595686

[77] Willcox BJ, Donlon TA, He Q, Chen R, Grove JS, Yano K, et al. FOXO3A genotype is strongly associated with human longevity. Proc Natl Acad Sci U S A. 2008;105. doi: 10.1073/pnas.0801030105 18765803 PMC2544566

[78] Morris BJ, Willcox BJ, Donlon TA. Genetic and epigenetic regulation of human aging and longevity. Biochim Biophys Acta Mol Basis Dis. 2019;1865:1718–1744. doi: 10.1016/j.bbadis.2018.08.039 31109447 PMC7295568

[79] Smeland OB, Wang Y, Frei O, Li W, Hibar DP, Franke B, et al. Genetic Overlap between Schizophrenia and Volumes of Hippocampus, Putamen, and Intracranial Volume Indicates Shared Molecular Genetic Mechanisms. Schizophr Bull. 2018;44. doi: 10.1093/schbul/sbx148 29136250 PMC6007549

[80] Le Grand Q, Satizabal CL, Sargurupremraj M, Mishra A, Soumaré A, Laurent A, et al. Genomic Studies Across the Lifespan Point to Early Mechanisms Determining Subcortical Volumes. Biol Psychiatry Cogn Neurosci Neuroimaging. 2022;7. doi: 10.1016/j.bpsc.2021.10.011 34700051 PMC9395126

[81] Luo X, Mao Q, Shi J, Wang X, Li C-SR. Putamen gray matter volumes in neuropsychiatric and neurodegenerative disorders. World J Psychiatry Ment Health Res. 2019;3. 31328186 PMC6641567

[82] Morey RA, Davis SL, Garrett ME, Haswell CC, Marx CE, Beckham JC, et al. Genome-wide association study of subcortical brain volume in PTSD cases and trauma-exposed controls. Transl Psychiatry. 2017;7. doi: 10.1038/s41398-017-0021-6 29187748 PMC5802459

[83] García-Marín LM, Reyes-Pérez P, Diaz-Torres S, Medina-Rivera A, Martin NG, Mitchell BL, et al. Shared molecular genetic factors influence subcortical brain morphometry and Parkinson’s disease risk. NPJ Parkinsons Dis. 2023;9. doi: 10.1038/s41531-023-00515-y 37164954 PMC10172359

[84] Chen CH, Wang Y, Lo MT, Schork A, Fan CC, Holland D, et al. Leveraging genome characteristics to improve gene discovery for putamen subcortical brain structure. Sci Rep. 2017;7. doi: 10.1038/s41598-017-15705-x 29147026 PMC5691156

[85] Sarnowski C, Ghanbari M, Bis JC, Logue M, Fornage M, Mishra A, et al. Meta-analysis of genome-wide association studies identifies ancestry-specific associations underlying circulating total tau levels. Commun Biol. 2022;5. doi: 10.1038/s42003-022-03287-y 35396452 PMC8993877

[86] Bowles KR, Pugh DA, Liu Y, Patel T, Renton AE, Bandres-Ciga S, et al. 17q21.31 sub-haplotypes underlying H1-associated risk for Parkinson’s disease are associated with LRRC37A/2 expression in astrocytes. Mol Neurodegener. 2022;17. doi: 10.1186/s13024-022-00551-x 35841044 PMC9284779

[87] Unlu G, Gamazon ER, Qi X, Levic DS, Bastarache L, Denny JC, et al. GRIK5 Genetically Regulated Expression Associated with Eye and Vascular Phenomes: Discovery through Iteration among Biobanks, Electronic Health Records, and Zebrafish. Am J Hum Genet. 2019;104:503–519. doi: 10.1016/j.ajhg.2019.01.017 30827500 PMC6407495

[88] Colbran LL, Gamazon ER, Zhou D, Evans P, Cox NJ, Capra JA. Inferred divergent gene regulation in archaic hominins reveals potential phenotypic differences. Nat Ecol Evol. 2019;3. doi: 10.1038/s41559-019-0996-x 31591491 PMC7046098

[89] Okamoto M, Inoue K, Iwamura H, Terashima K, Soya H, Asashima M, et al. Reduction in paracrine Wnt3 factors during aging causes impaired adult neurogenesis. FASEB J. 2011;25. doi: 10.1096/fj.11-184697 21746862

[90] Duan RS, Liu PP, Xi F, Wang WH, Tang GB, Wang RY, et al. Wnt3 and Gata4 regulate axon regeneration in adult mouse DRG neurons. Biochem Biophys Res Commun. 2018;499. doi: 10.1016/j.bbrc.2018.03.138 29567480

[91] Wang D, Wu J. A novel variant in the QRICH1 gene was identified in a patient with severe developmental delay. Mol Genet Genomic Med. 2023;11. doi: 10.1002/mgg3.2227 37331002 PMC10422060

[92] Kumble S, Levy AM, Punetha J, Gao H, Ah Mew N, Anyane-Yeboa K, et al. The clinical and molecular spectrum of QRICH1 associated neurodevelopmental disorder. Hum Mutat. 2022;43. doi: 10.1002/humu.24308 34859529

[93] Bost DM, Bizon C, Tilson JL, Filer DL, Gizer IR, Wilhelmsen KC. Association of Predicted Expression and Multimodel Association Analysis of Substance Abuse Traits. Complex Psychiatry. 2022:8. doi: 10.1159/000523748 36407771 PMC9669989

[94] Ancelin M-L, Norton J, Ritchie K, Chaudieu I, Ryan J. Steroid 21-hydroxylase gene variants and late-life depression. BMC Res Notes. 2021;14:203. doi: 10.1186/s13104-021-05616-6 34034803 PMC8147346

[95] Kamran M, Bibi F, Ur Rehman A, Morris DW. Major depressive disorder: Existing hypotheses about pathophysiological mechanisms and new genetic findings. Genes (Basel). 2022;13:646. doi: 10.3390/genes13040646 35456452 PMC9025468

[96] Bahrami S, Nordengen K, Shadrin AA, Frei O, van der Meer D, Dale AM, et al. Distributed genetic architecture across the hippocampal formation implies common neuropathology across brain disorders. Nat Commun. 2022:13. doi: 10.1038/s41467-022-31086-w 35705537 PMC9200849

[97] Roelfs D, Frei O, van der Meer D, Tissink E, Shadrin A, Alnaes D, et al. Shared genetic architecture between mental health and the brain functional connectome in the UK Biobank. BMC Psychiatry. 2023:23. doi: 10.1186/s12888-023-04905-7 37353766 PMC10290393

[98] Attfield KE, Jensen LT, Kaufmann M, Friese MA, Fugger L. The immunology of multiple sclerosis. Nat Rev Immunol. 2022;22:734–750. doi: 10.1038/s41577-022-00718-z 35508809

[99] Hamza TH, Zabetian CP, Tenesa A, Laederach A, Montimurro J, Yearout D, et al. Common genetic variation in the HLA region is associated with late-onset sporadic Parkinson’s disease. Nat Genet. 2010:42. doi: 10.1038/ng.642 20711177 PMC2930111

[100] Diamond A. Close interrelation of motor development and cognitive development and of the cerebellum and prefrontal cortex. Child Dev. 2000;71. doi: 10.1111/1467-8624.00117 10836557

[101] de Rooij AM, Florencia Gosso M, Haasnoot GW, Marinus J, Verduijn W, Claas FHJ, et al. HLA-B62 and HLA-DQ8 are associated with Complex Regional Pain Syndrome with fixed dystonia. Pain. 2009;145. doi: 10.1016/j.pain.2009.05.015 19523767

[102] Kerestes R, Laansma MA, Owens-Walton C, Perry A, van Heese EM, Al-Bachari S, et al. Cerebellar Volume and Disease Staging in Parkinson’s Disease: An ENIGMA-PD Study. Mov Disord. 2023. doi: 10.1002/mds.29611 37964373 PMC10754393

[103] Ahn EH, Kang SS, Qi Q, Liu X, Ye K. Netrin1 deficiency activates MST1 via UNC5B receptor, promoting dopaminergic apoptosis in Parkinson’s disease. Proc Natl Acad Sci U S A. 2020;117. doi: 10.1073/pnas.2004087117 32929029 PMC7533679

[104] Tian Y, Ma G, Li H, Zeng Y, Zhou S, Wang X, et al. Shared Genetics and Comorbid Genes of Amyotrophic Lateral Sclerosis and Parkinson’s Disease. Mov Disord. 2023. doi: 10.1002/mds.29572 37534731

[105] Azevedo C, Teku G, Pomeshchik Y, Reyes JF, Chumarina M, Russ K, et al. Parkinson’s disease and multiple system atrophy patient iPSC-derived oligodendrocytes exhibit alpha-synuclein–induced changes in maturation and immune reactive properties. Proc Natl Acad Sci U S A. 2022:119. doi: 10.1073/pnas.2111405119 35294277 PMC8944747

[106] Ranlund S, Rosa MJ, de Jong S, Cole JH, Kyriakopoulos M, Fu CHY, et al. Associations between polygenic risk scores for four psychiatric illnesses and brain structure using multivariate pattern recognition. Neuroimage Clin. 2018;20:1026–1036. doi: 10.1016/j.nicl.2018.10.008 30340201 PMC6197704

[107] Alnæs D, Kaufmann T, van der Meer D, Córdova-Palomera A, Rokicki J, Moberget T, et al. Brain Heterogeneity in Schizophrenia and Its Association With Polygenic Risk. JAMA Psychiatry. 2019;76:739–748. doi: 10.1001/jamapsychiatry.2019.0257 30969333 PMC6583664

[108] Torkamani A, Wineinger NE, Topol EJ. The personal and clinical utility of polygenic risk scores. Nat Rev Genet. 2018;19:581–590. doi: 10.1038/s41576-018-0018-x 29789686

[109] Yang H, Long X-Y, Yang Y, Yan H, Zhu C-Z, Zhou X-P, et al. Amplitude of low frequency fluctuation within visual areas revealed by resting-state functional MRI. Neuroimage. 2007;36:144–152. doi: 10.1016/j.neuroimage.2007.01.054 17434757

[110] Jiang L, Zuo X-N. Regional homogeneity: a multimodal, multiscale neuroimaging marker of the human connectome. Neuroscientist. 2016;22:486–505. doi: 10.1177/1073858415595004 26170004 PMC5021216

[111] Cole MW, Pathak S, Schneider W. Identifying the brain’s most globally connected regions. Neuroimage. 2010;49:3132–3148. doi: 10.1016/j.neuroimage.2009.11.001 19909818

[112] Sainburg LE, Little AA, Johnson GW, Janson AP, Levine KK, González HFJ, et al. Characterization of resting functional MRI activity alterations across epileptic foci and networks. Cereb Cortex. 2022. doi: 10.1093/cercor/bhac035 35149867 PMC9753043

[113] Liu Y, Wang K, Yu C, He Y, Zhou Y, Liang M, et al. Regional homogeneity, functional connectivity and imaging markers of Alzheimer’s disease: A review of resting-state fMRI studies. Neuropsychologia. 2008;46. doi: 10.1016/j.neuropsychologia.2008.01.027 18346763

[114] Shang CY, Lin HY, Tseng WY, Gau SS. A haplotype of the dopamine transporter gene modulates regional homogeneity, gray matter volume, and visual memory in children with attention-deficit/hyperactivity disorder. Psychol Med. 2018;48. doi: 10.1017/S0033291718000144 29433615

[115] Sullivan GM, Feinn R. Using Effect Size—or Why the P Value Is Not Enough. J Grad Med Educ. 2012;4:279–282. doi: 10.4300/JGME-D-12-00156.1 23997866 PMC3444174

[116] Lazar C, Meganck S, Taminau J, Steenhoff D, Coletta A, Molter C, et al. Batch effect removal methods for microarray gene expression data integration: a survey. Brief Bioinform. 2013;14:469–490. doi: 10.1093/bib/bbs037 22851511

[117] Davey Smith G, Ebrahim S. ‘Mendelian randomization’: can genetic epidemiology contribute to understanding environmental determinants of disease?*. Int J Epidemiol. 2003;32:1–22. doi: 10.1093/ije/dyg070 12689998

[118] Zhu X. Mendelian randomization and pleiotropy analysis. Quant Biol. 2021;9:122–132. doi: 10.1007/s40484-020-0216-3 34386270 PMC8356909

[119] Smith GD, Ebrahim S. Mendelian randomisation at 20 years: how can it avoid hubris, while achieving more? Lancet Diabetes Endocrinol. 2023. doi: 10.1016/S2213-8587(23)00348-0 38048796

[120] Horien C, Greene AS, Constable RT, Scheinost D. Regions and Connections: Complementary Approaches to Characterize Brain Organization and Function. Neuroscientist. 2019;26:117–133. doi: 10.1177/1073858419860115 31304866 PMC7079335

[121] Hawrylycz M, Martone ME, Ascoli GA, Bjaalie JG, Dong H-W, Ghosh SS, et al. A guide to the BRAIN Initiative Cell Census Network data ecosystem. PLoS Biol. 2023;21:e3002133. doi: 10.1371/journal.pbio.3002133 37390046 PMC10313015

[122] Carithers LJ, Ardlie K, Barcus M, Branton PA, Britton A, Buia SA, et al. A Novel Approach to High-Quality Postmortem Tissue Procurement: The GTEx Project. Biopreserv Biobank. 2015;13:311–319. doi: 10.1089/bio.2015.0032 26484571 PMC4675181

[123] Stegle O, Parts L, Piipari M, Winn J, Durbin R. Using probabilistic estimation of expression residuals (PEER) to obtain increased power and interpretability of gene expression analyses. Nat Protoc. 2012;7:500–507. doi: 10.1038/nprot.2011.457 22343431 PMC3398141

[124] Pauli WM, Nili AN, Tyszka JM. A high-resolution probabilistic in vivo atlas of human subcortical brain nuclei. Sci Data. 2018;5:180063. doi: 10.1038/sdata.2018.63 29664465 PMC5903366

[125] Diedrichsen J, Balsters JH, Flavell J, Cussans E, Ramnani N. A probabilistic MR atlas of the human cerebellum. Neuroimage. 2009;46:39–46. doi: 10.1016/j.neuroimage.2009.01.045 19457380

[126] Jenkinson M, Beckmann CF, Behrens TEJ, Woolrich MW, Smith SM. FSL. Neuroimage. 2012;62:782–790. doi: 10.1016/j.neuroimage.2011.09.015 21979382

[127] Klein A, Tourville J. 101 Labeled Brain Images and a Consistent Human Cortical Labeling Protocol. Front Neurosci. 2012;6. doi: 10.3389/fnins.2012.00171 23227001 PMC3514540

[128] Desikan RS, Ségonne F, Fischl B, Quinn BT, Dickerson BC, Blacker D, et al. An automated labeling system for subdividing the human cerebral cortex on MRI scans into gyral based regions of interest. Neuroimage. 2006;31:968–980. doi: 10.1016/j.neuroimage.2006.01.021 16530430

[129] Fischl B, Salat DH, Busa E, Albert M, Dieterich M, Haselgrove C, et al. Whole Brain Segmentation: Automated Labeling of Neuroanatomical Structures in the Human Brain. Neuron. 2002;33:341–355. doi: 10.1016/s0896-6273(02)00569-x 11832223

[130] Hawrylycz MJ, Lein ES, Guillozet-Bongaarts AL, Shen EH, Ng L, Miller JA, et al. An anatomically comprehensive atlas of the adult human brain transcriptome. Nature. 2012;489:391. doi: 10.1038/nature11405 22996553 PMC4243026

[131] Markello RD, Arnatkeviciute A, Poline J-B, Fulcher BD, Fornito A, Misic B. Standardizing workflows in imaging transcriptomics with the abagen toolbox. Elife. 2021;10:e72129. doi: 10.7554/eLife.72129 34783653 PMC8660024

[132] Gorgolewski KJ, Fox AS, Chang L, Schäfer A, Arélin K, Burmann I, et al. Tight fitting genes: finding relations between statistical maps and gene expression patterns. F1000Res. 2014;5.

[133] Alfaro-Almagro F, Jenkinson M, Bangerter NK, Andersson JLR, Griffanti L, Douaud G, et al. Image processing and Quality Control for the first 10,000 brain imaging datasets from UK Biobank. Neuroimage. 2018;166:400–424. doi: 10.1016/j.neuroimage.2017.10.034 29079522 PMC5770339

[134] McCarthy S, Das S, Kretzschmar W, Delaneau O, Wood AR, Teumer A, et al. A reference panel of 64,976 haplotypes for genotype imputation. Nat Genet. 2016;48. doi: 10.1038/ng.3643 27548312 PMC5388176

[135] Huang J, Howie B, McCarthy S, Memari Y, Walter K, Min JL, et al. Improved imputation of low-frequency and rare variants using the UK10K haplotype reference panel. Nat Commun. 2015;6. doi: 10.1038/ncomms9111 26368830 PMC4579394

[136] Van Essen DC, Ugurbil K, Auerbach E, Barch D, Behrens TEJ, Bucholz R, et al. The Human Connectome Project: a data acquisition perspective. Neuroimage. 2012;62:2222–2231. doi: 10.1016/j.neuroimage.2012.02.018 22366334 PMC3606888

[137] Glasser MF, Sotiropoulos SN, Wilson JA, Coalson TS, Fischl B, Andersson JL, et al. The minimal preprocessing pipelines for the Human Connectome Project. Neuroimage. 2013;80:105–124. doi: 10.1016/j.neuroimage.2013.04.127 23668970 PMC3720813

[138] Marcus DS, Harms MP, Snyder AZ, Jenkinson M, Wilson JA, Glasser MF, et al. Human Connectome Project informatics: Quality control, database services, and data visualization. Neuroimage. 2013;80:202–219. doi: 10.1016/j.neuroimage.2013.05.077 23707591 PMC3845379

[139] Salimi-Khorshidi G, Douaud G, Beckmann CF, Glasser MF, Griffanti L, Smith SM. Automatic denoising of functional MRI data: Combining independent component analysis and hierarchical fusion of classifiers. Neuroimage. 2014;90:449–468. doi: 10.1016/j.neuroimage.2013.11.046 24389422 PMC4019210

[140] Price AL, Patterson NJ, Plenge RM, Weinblatt ME, Shadick NA, Reich D. Principal components analysis corrects for stratification in genome-wide association studies. Nat Genet. 2006;38:904–909. doi: 10.1038/ng1847 16862161

[141] Robinson EC, Garcia K, Glasser MF, Chen Z, Coalson TS, Makropoulos A, et al. Multimodal surface matching with higher-order smoothness constraints. Neuroimage. 2018;167:453–465. doi: 10.1016/j.neuroimage.2017.10.037 29100940 PMC5991912

[142] Liu TT, Nalci A, Falahpour M. The global signal in fMRI: Nuisance or Information? Neuroimage. 2017;150:213–229. doi: 10.1016/j.neuroimage.2017.02.036 28213118 PMC5406229

[143] Mbatchou J, Barnard L, Backman J, Marcketta A, Kosmicki JA, Ziyatdinov A, et al. Computationally efficient whole-genome regression for quantitative and binary traits. Nat Genet. 2021;53:1097–1103. doi: 10.1038/s41588-021-00870-7 34017140

[144] Bastarache L. Using Phecodes for research with the electronic health record: from PheWAS to PheRS. Annu Rev Biomed Data Sci. 2021;4:1–19. doi: 10.1146/annurev-biodatasci-122320-112352 34465180 PMC9307256

[145] Zhang B, Kirov S, Snoddy J. WebGestalt: An integrated system for exploring gene sets in various biological contexts. Nucleic Acids Res. 2005:33. doi: 10.1093/nar/gki475 15980575 PMC1160236

[146] Liao Y, Wang J, Jaehnig EJ, Shi Z, Zhang B. WebGestalt 2019: gene set analysis toolkit with revamped UIs and APIs. Nucleic Acids Res. 2019;47. doi: 10.1093/nar/gkz401 31114916 PMC6602449

