# Supplementary figures and images for: Integration of estimated regional gene expression with neuroimaging and clinical phenotypes at biobank scale
Source: PLoS Biol. 2024 Sep 13;22(9):e3002782. doi: 10.1371/journal.pbio.3002782 (PMC11424006; doi:10.1371/journal.pbio.3002782)

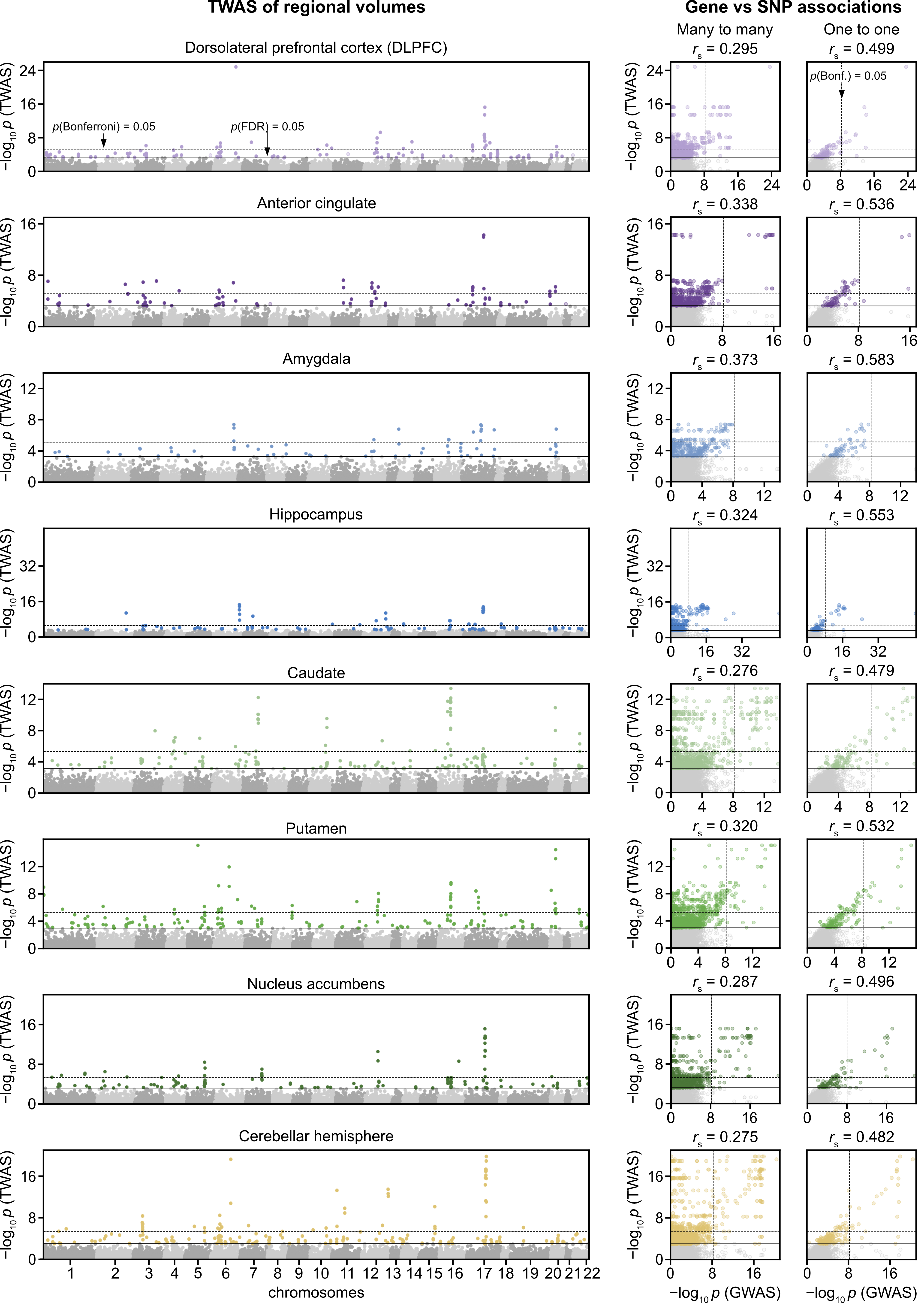

Supplement: S1 Fig — TWAS of gr-expression and brain volumes for all regions. Each point denotes an association between the individual variation of gr-expression of a gene and volume in the same region. The horizontal axis denotes the chromosome location of individual genes. The vertical axis denotes–log10 p-values. Solid-color points represent associations that passed pFDR = 0.05 or pBonferroni = 0.05 (horizontal lines). Right. Associations between SNP-based GWAS and gene-based TWAS for all regions. Left: Scatter plots of p-values (–log10 p) for associations of all genes and SNPs. These plots preserve all genes and SNPs but lack the one-to-one relationship between genes and SNPs. Right: Corresponding scatter plots of the best-performing genes and SNPs. Each gene in TWAS matches with its best-performing SNP in GWAS. Similarly, each SNP in GWAS matches with its best-performing gene in TWAS. These plots show one-to-one relationships but exclude many genes and SNPs. (TIFF) [file pbio.3002782.s001.tiff]

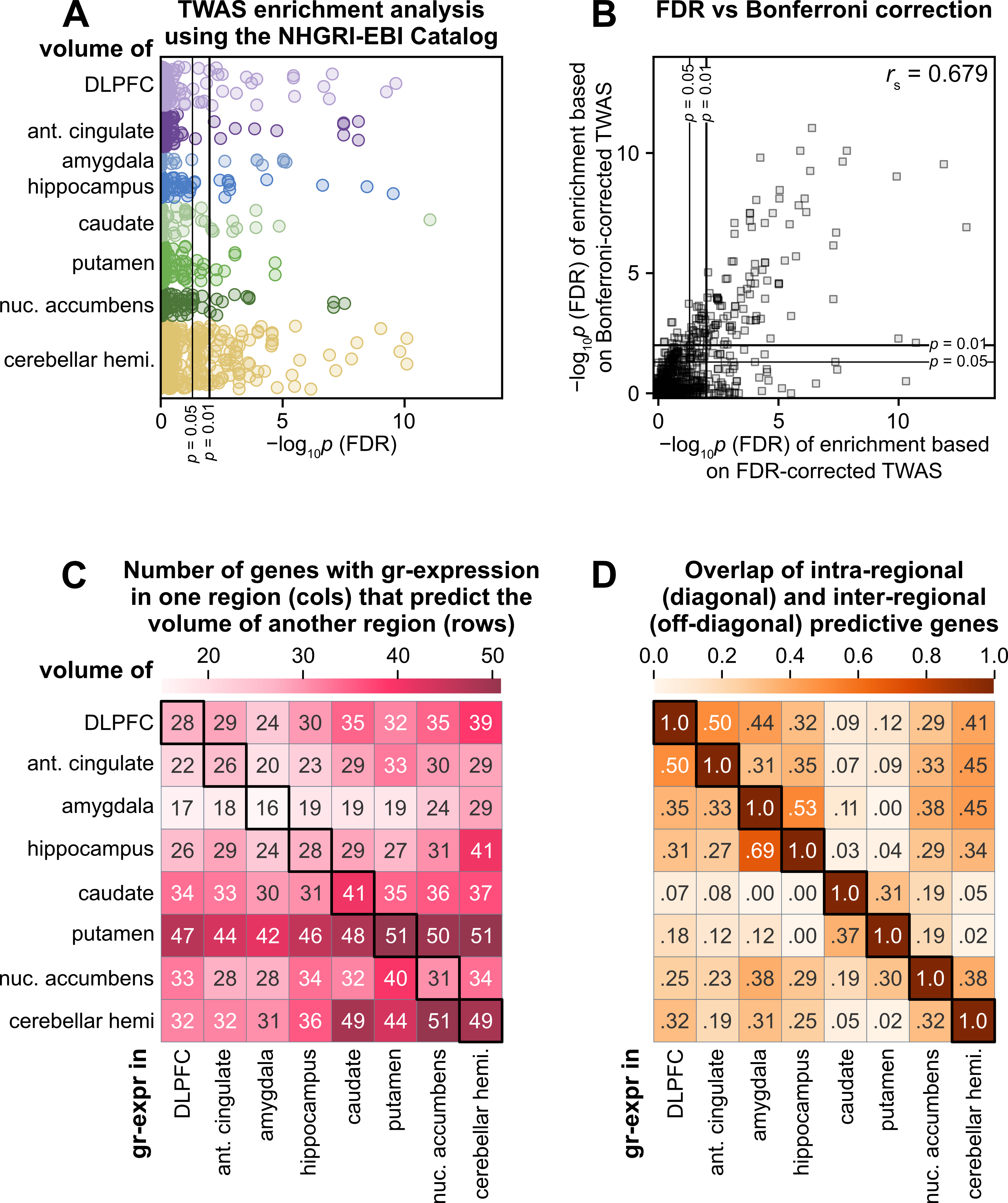

Supplement: S2 Fig — (A, B) Effects of Bonferroni correction on enrichment analyses in the NHGRI-EBI Catalog. (A) Enrichment for biological annotations of genes whose gr-expression predicted regional volumes (pBonferroni < 0.05). Each point represents a biological annotation associated with at least 1 gene. The horizontal axis denotes the p-values (–log10 pFDR) of individual annotations. (B) Comparison of pFDR for biological annotations of genes whose gr-expression predicted regional volumes under FDR and Bonferroni corrections. (C, D) Effects of Bonferroni correction on inter-regional associations between gr-expression and regional brain volumes. (C) Absolute numbers of associations. Numbers of genes whose gr-expression in one region (columns) predicted (pBonferroni < 0.05) the volume of another region (rows). (D) Overlap coefficients. Number of genes that were common to both intra-regional and inter-regional associations in C, normalized by the size of the smaller of the intra- and inter-regional gene sets. (TIFF) [file pbio.3002782.s002.tiff]

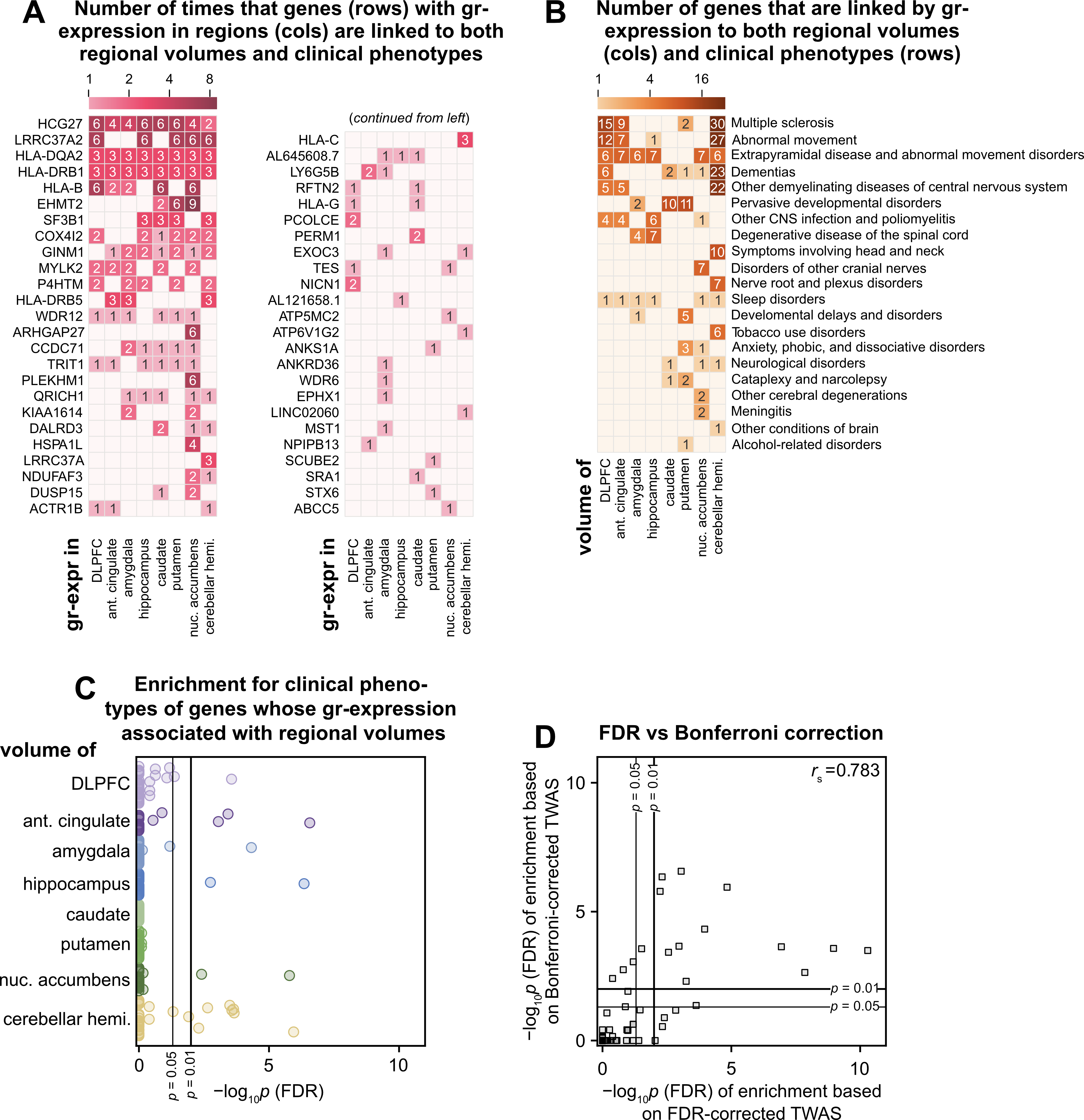

Supplement: S3 Fig — (A) Heatmap showing the number of times by which genes (rows) with regional gr-expression (columns) were linked to both regional volumes and clinical phenotypes. Each count denotes a regional gr-expression that was associated with both a regional volume in the UK Biobank TWAS and with a brain-related clinical phenotype in the BioVU TWAS (pBonferroni < 0.05). (B) Heatmap showing the number of genes with regional gr-expression that linked regional volumes (columns) with clinical phenotypes (rows). Each count denotes a regional gr-expression that was associated with both a regional volume in the UK Biobank TWAS and with a brain-related clinical phenotype in the BioVU TWAS (pBonferroni < 0.05). (C) Enrichment of clinical phenotypes for genes whose gr-expression predicted regional volumes (rows) in the UK Biobank TWAS (pBonferroni < 0.05). Each point represents a brain-related clinical phenotype associated with at least 1 gene. The horizontal axis denotes the p-values (–log10 pFDR) of individual phenotypes. (D) Comparison of pFDR for clinical phenotypes of genes whose gr-expression predicted regional volumes under FDR and Bonferroni corrections. (TIFF) [file pbio.3002782.s003.tiff]

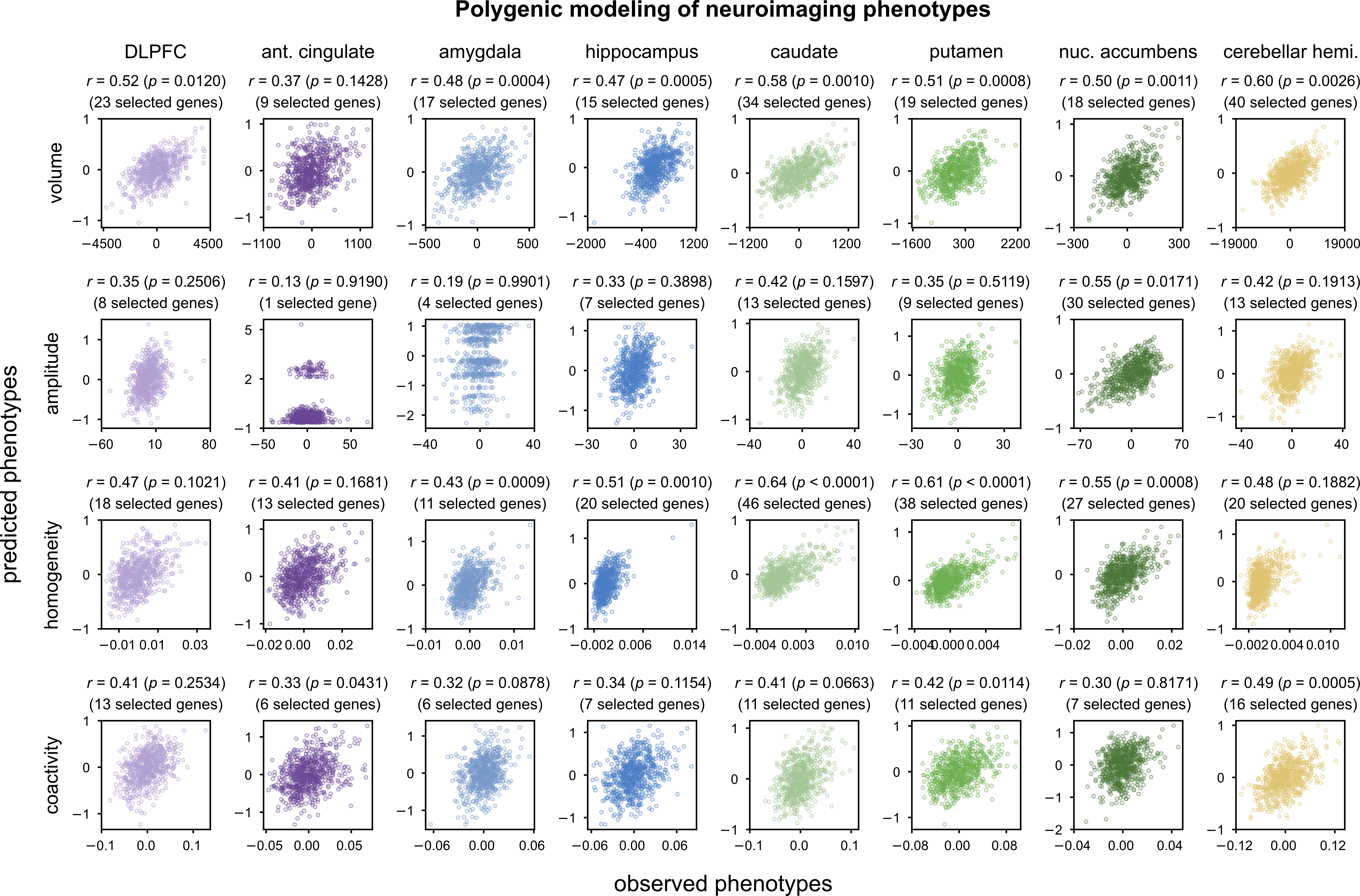

Supplement: S4 Fig — Scatter plots of polygenic gr-expression and neuroimaging phenotypes. The horizontal axis shows values of observed phenotypes, and the vertical axis denotes values of polygenic gr-expression. Points represent single individuals. (TIFF) [file pbio.3002782.s004.tiff]

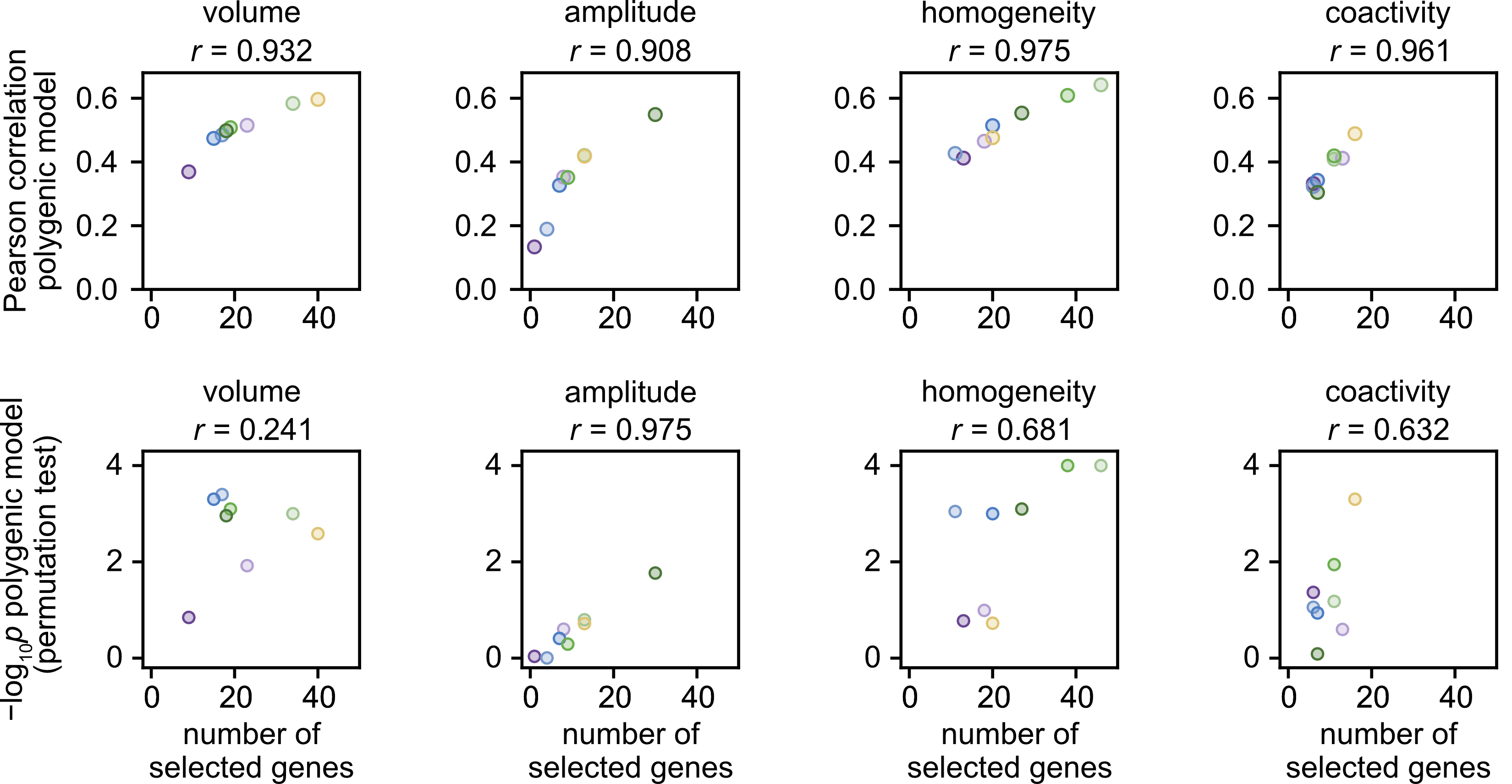

Supplement: S5 Fig — Scatter plots showing the number of genes in each polygenic model and model r-values and p-values (–log10 pFDR from permutation testing). Each plot shows a distinct phenotype. Colors denote brain regions as in Fig 5. (TIFF) [file pbio.3002782.s005.tiff]

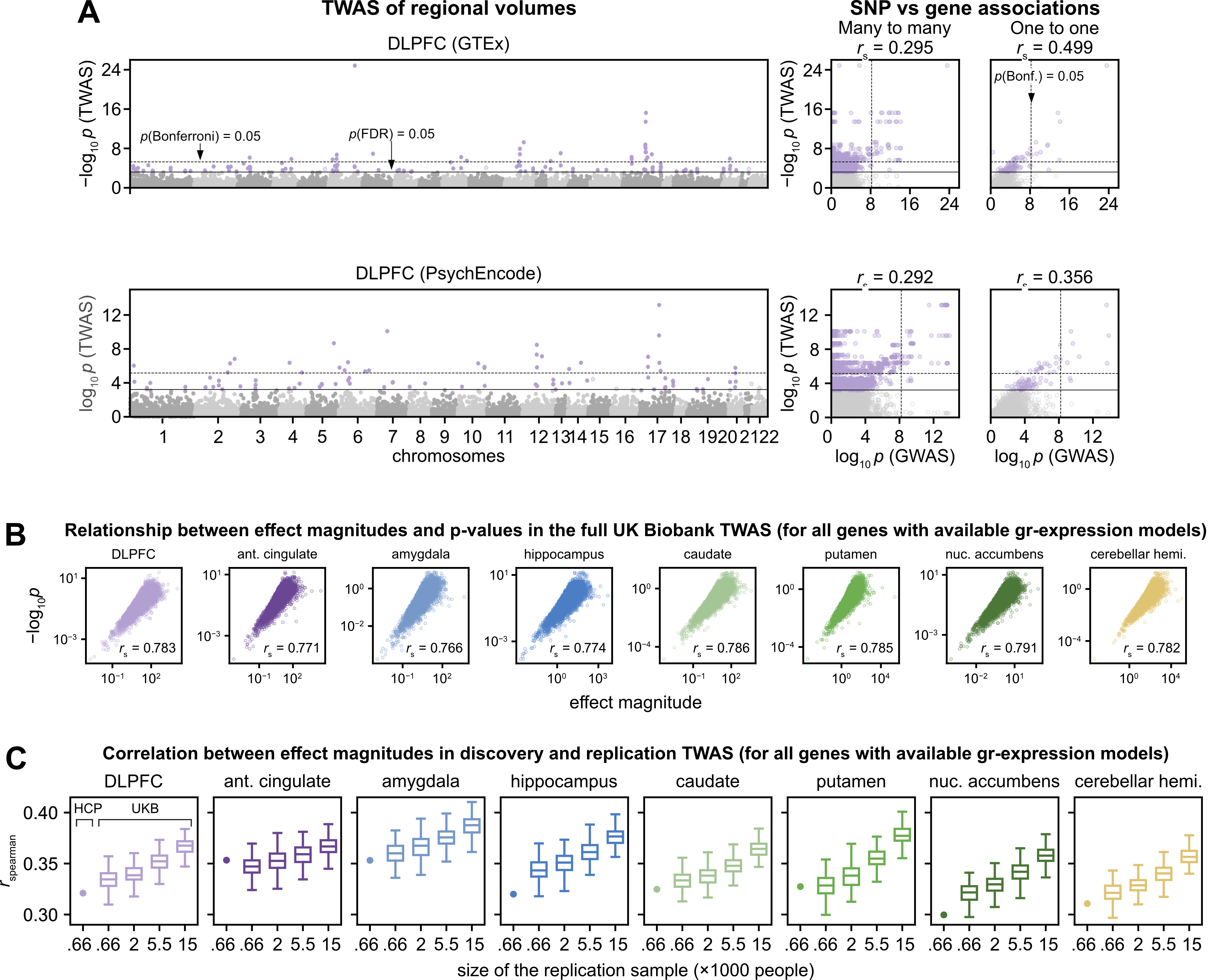

Supplement: S6 Fig — (A) Left. Within-regional associations of gr-expression and gray-matter volumes for the DLPFC, based on gr-expression models trained on GTEx and PsychEncode data. Each point denotes an association between the individual variation of gr-expression and volume in the same region. The horizontal axis shows the chromosome location of individual genes. The vertical axis shows the p-values (–log10 p) of associations. Solid-color points show associations that passed pFDR = 0.05 or pBonferroni = 0.05 (horizontal lines). (A) Right. Associations between SNP-based GWAS and gene-based TWAS for the DLPFC, based on gr-expression models trained on GTEx and PsychEncode data. Left: Scatter plots of p-values (–log10 p) for associations of all genes and SNPs. These plots preserve all genes and SNPs but lack the one-to-one relationship between genes and SNPs. Right: Corresponding scatter plots for the best-performing genes and SNPs. Each gene in TWAS matches with its best-performing SNP in GWAS. Similarly, each SNP in GWAS matches with its best-performing gene in TWAS. These plots show one-to-one relationships but exclude many genes and SNPs. (B) Scatter plots of effect magnitudes and p-values (–log10 p) for the UK Biobank TWAS of regional gray-matter volumes. Dots denote associations for all genes from the TWAS. Note the double-logarithmic scale. (C) Correlations between effect magnitudes of all gene associations in the replication and discovery TWAS of regional gray-matter volumes. Dots denote analyses on the full UK Biobank (discovery) and HCP (replication) samples. Box plots denote analyses of discovery-replication splits of the white-British UK-Biobank sample, ordered from small to large replication samples. Each box plot was estimated from 300 random splits of the white-British UK-Biobank sample. Fig 6D shows a similar plot, but filtered to include only genes that passed pFDR < 0.05 in the discovery TWAS. (TIFF) [file pbio.3002782.s006.tiff]
